# Acute Hypoxia Induces Transient Olfactory Dysfunction through Olfactory Epithelial Degeneration and Bulbar Mitochondrial Stress in Zebrafish

**DOI:** 10.1523/ENEURO.0119-26.2026

**Published:** 2026-07-28

**Authors:** Skylar L. DeWitt-Batt, Kate E. DeMann, Cameron J. Houck, Cassidy L. Larson, Luke A. Horsburgh, Evan A. Thomas, Liz Sanchez, Erika Calvo-Ochoa

**Affiliations:** ^1^Biology Department and Neuroscience Department, Hope College, Holland, Michigan 49423; ^2^Department of Neuroscience, University of Rochester Medical Center, Rochester, New York 14642

**Keywords:** hypoxia, ischemia, neurodegeneration, neurogenesis, neuroinflammation, olfactory dysfunction; olfactory system, zebrafish

## Abstract

Hypoxic–ischemic injury is a major cause of olfactory dysfunction, yet the cellular and morphological mechanisms underlying this sensory loss remain poorly understood. Here, we investigated the structural, cellular, and functional effects of acute hypoxic exposure on the olfactory system of adult zebrafish (*Danio rerio*) of both sexes, a model organism with remarkable neuroregenerative capacity. Fish were subjected to 15 min of acute severe hypoxia (0.8 mg/L DO) and assessed at 1 and 5 d posthypoxia. We evaluated olfactory function by means of cadaverine-evoked aversive behavioral assays. Structural and morphological integrity and inflammation of the olfactory epithelium (OE) and olfactory bulb (OB) were characterized using immunohistochemistry, histological stainings, and a 2,3,5-triphenyltetrazolium chloride colorimetric assay. Acute hypoxic exposure impaired olfactory-mediated behaviors without affecting locomotion or exploratory behavior. In the peripheral OE, hypoxia caused neurodegeneration, disruption of the nasal mucus layer, and robust leukocytic infiltration. We observed reduced mitochondrial dehydrogenase activity in the OB along with reactive astrogliosis. Olfactory function recovered by 5 d, coinciding with full restoration of OE morphology, which was supported by a strong proliferative response. These findings reveal a coordinated degenerative and regenerative response to hypoxia across the olfactory axis, with implications for understanding hypoxia-induced sensory loss and neural repair.

## Graphical abstract

**Figure:**
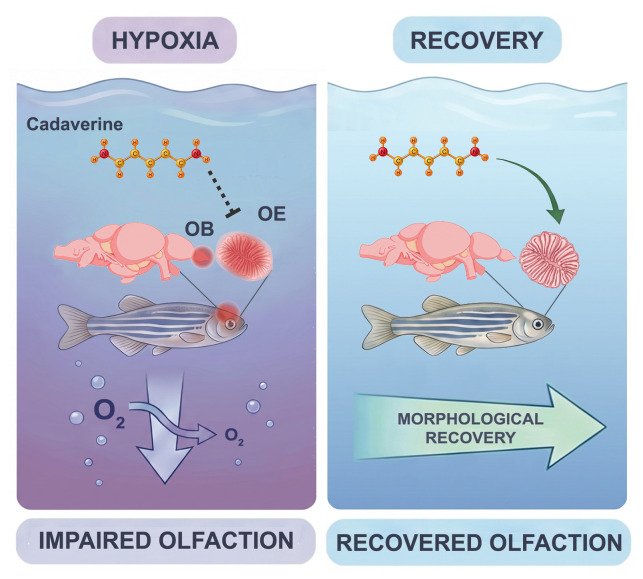
Visual Abstract

## Significance Statement

This work addresses an important gap in knowledge regarding the mechanisms linking hypoxic insult and olfactory dysfunction. By using adult zebrafish, an extraordinarily regenerative vertebrate, it also provides insight into neuronal repair and regenerative processes supporting olfactory recovery. The novelty of our study resides in that, to our knowledge, there are no studies that provide a comprehensive characterization of the effects of hypoxia in the olfactory system across molecular, histological, and functional levels. These findings advance our understanding of hypoxia-induced sensory neurodegeneration and regeneration and highlight the zebrafish olfactory system as a powerful model for investigating neural repair mechanisms relevant to hypoxic–ischemic brain injury.

## Introduction

Tissue hypoxia, defined as insufficient oxygen in tissues to sustain physiological functions, poses a significant threat to terrestrial and aquatic organisms. In clinical settings, hypoxia arises from conditions including ischemic stroke (Leu et al., 2019; Feng et al., 2020) and traumatic brain injury (Yan et al., 2014; Scultetus et al., 2016), among others. The brain is particularly vulnerable to oxygen deprivation, as neurons rely on oxidative phosphorylation to meet their extensive energetic demands (Busl and Greer, 2010). Hypoxic exposure can compromise mitochondrial function and integrity due to increased oxidative stress, reduced ATP production, and altered cellular metabolism (Douglas et al., 2010; Dhillon and Richards, 2018), leading to a broad spectrum of sensory and cognitive deficits (Lee et al., 2018; Hernández-Soto et al., 2021; Wang et al., 2022).

Although olfactory loss is a well-recognized consequence of hypoxic–ischemic brain injury in mammals (Bigdai et al., 2018; Huppertz et al., 2018), the mechanisms underlying its onset and recovery are poorly understood, given the limited regenerative capacity of the mammalian nervous system (Kaslin et al., 2008). Addressing this gap necessitates a model that enables investigation of both degenerative and regenerative processes following hypoxic insult. The olfactory system of adult zebrafish offers such a model, given its unique and well-characterized regenerative and neurogenic capabilities, supported by progenitor cell populations across the olfactory axis (Calvo-Ochoa et al., 2019; Calvo-Ochoa et al., 2021) and at the adjacent telencephalon that respond rapidly to injury and promote structural repair (Adolf et al., 2006; Marz et al., 2010). Injury-induced neuroinflammation, mediated by resident neutrophils and recruited macrophages, is also a well-characterized component of peripheral olfactory repair in zebrafish (Palominos et al., 2022). Furthermore, olfactory-mediated behavioral assays allow functional outcomes to be directly linked to morphological states across recovery timepoints (Calvo-Ochoa et al., 2025; Vorhees et al., 2025).

The zebrafish olfactory system is similar to those of mammals, comprising three components across the peripheral and central nervous system (Byrd and Brunjes, 1995; Saraiva et al., 2015). The olfactory epithelium (OE), located in the olfactory cavity, contains olfactory sensory neurons (OSNs) that detect odorants via specialized cilia extending into the mucus layer (Hansen and Zielinski, 2005; Yoshihara, 2009). OSNs transmit sensory signals through the olfactory nerve (ON), a short bundle of axons wrapped by olfactory ensheathing cells (OECs) traversing the ethmoid bone to the olfactory bulb (OB), located immediately posterior at the rostral end of the telencephalon (Mori et al., 1999; Sato et al., 2005; Lazzari et al., 2014) where signals are relayed to higher brain centers (Miyasaka et al., 2009).

The olfactory system is uniquely vulnerable to low-oxygen conditions given that the OE is directly exposed to the aquatic environment and is susceptible to changes in water chemistry, including reduced oxygen levels (Williams et al., 2019; Simonis et al., 2024). Furthermore, the OE lacks vascularization, meaning OSNs rely entirely on passive oxygen diffusion to meet their high energetic demands (Bigdai et al., 2019). While reports of the effects of hypoxia on brain function in zebrafish have started to emerge (Braga et al., 2013; Das et al., 2019; Park et al., 2021), its impact on the olfactory system remains largely unexplored. To our knowledge, only one study has directly examined hypoxia-induced olfactory dysfunction in fish (Tigert et al., 2025), and no study has investigated the mechanistic basis of this dysfunction.

Here, we aimed to investigate the functional and structural consequences of acute hypoxic insult to the olfactory system of adult zebrafish. We hypothesized that hypoxia would compromise the structural integrity of the OE and OB, leading to impaired olfactory function, and that the regenerative capacity of the zebrafish olfactory system would drive subsequent structural and functional recovery. To test this, we used a severe but sublethal acute hypoxic exposure paradigm (Marino et al., 2020) and assessed olfactory function and morphology at 1 and 5 d posthypoxia (dph)—timepoints selected based on prior reports demonstrating OE structural recovery by 5 d following chemical ablation of the OE (Iqbal and Byrd-Jacobs, 2010; Hentig and Byrd-Jacobs, 2016). We show that acute hypoxic exposure results in olfactory dysfunction associated with OE neurodegeneration, OB metabolic stress, and peripheral and central inflammation, followed by structural and functional recovery by 5 dph. These findings provide the first cellular and morphological framework for understanding hypoxia-induced olfactory dysfunction and recovery in a vertebrate model, setting the foundation for future studies dissecting the precise mechanisms and timepoints of repair across the olfactory axis.

## Materials and Methods

### Animals

Adult wild-type zebrafish (*Danio rerio)* of both sexes were kept and bred in an aquatic system at 28°C (Aquaneering) located at Hope College's zebrafish facility. The facility was kept on a 12 h light/dark cycle. Routine feedings consisted of pellet fish food (Reef Nutrition) twice daily and fresh brine shrimp (Brine Shrimp Direct) once daily. Adult wild-type zebrafish of both sexes from ∼6–18 months of age were used for all experiments. Control and hypoxia-treated fish were selected from the same housing tanks, exposed to their respective treatments in parallel, and processed concurrently. Experiments were repeated using multiple cohorts of fish collected over several months. All experimental procedures were performed in accordance with the National Institutes of Health Guide for the Care and Use of Laboratory Animals (NIH Publication No. 80-23) and were approved by Hope College's Institutional Animal Care and Use Committee. All efforts were made to minimize the number of animals used.

### Acute hypoxic exposure

**Figure 1.**
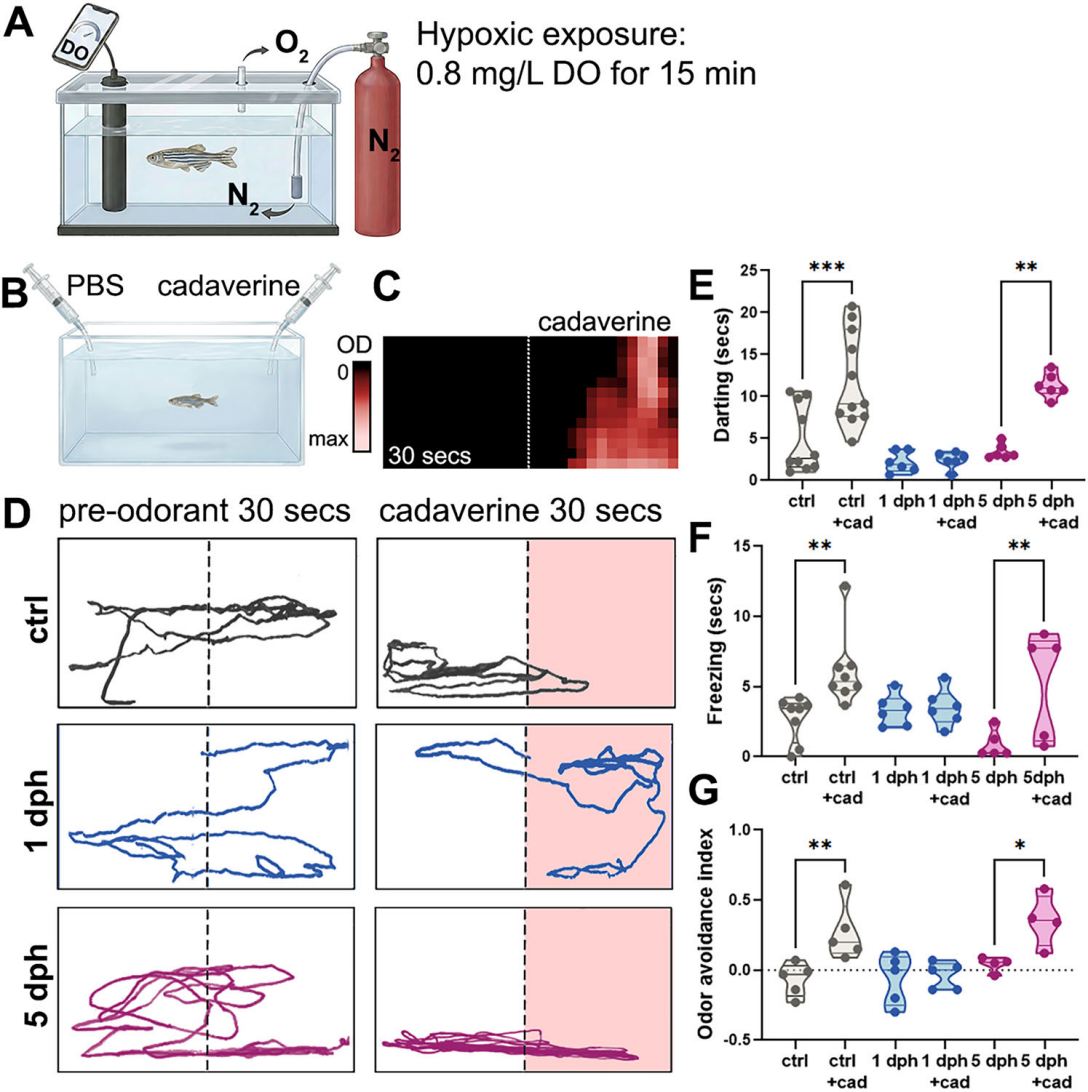
Odor-mediated behavioral responses to cadaverine in control and hypoxic fish. ***A***, Hypoxic exposure setup. We used a sealed hypoxic chamber with two ports. Nitrogen gas (N2) was delivered through a submerged inlet connected to an N2 cylinder into 300 ml of fish water. A second outlet tube allowed for excess gas exchange. DO levels were continuously monitored with a submerged digital DO probe. Once DO concentration reached 0.8 mg/L, nitrogen perfusion was stopped, and individual fish were placed in the sealed tank for 15 min. ***B***, Schematic of behavioral assay tank. PBS (control) and cadaverine (odorant) were dispensed into opposite sides of the tank. ***C***, Heatmap of cadaverine dispersal 30 s after delivery, indicating that cadaverine remains on one side of the behavioral tank. ***D***, Representative swimming trajectories of individual zebrafish 30 s pre- (left panels) and 30 s postcadaverine administration (right panels, shaded in pink) in controls (top panels), 1 dph (middle panel), and 5 dph (bottom panels) fish. ***E***, Time spent darting after cadaverine exposure in normoxic controls, 1 dph, and 5 dph fish. ctrl versus cad *p* = 0.0001; 5 versus 5 dph cad *p* = 0.0012 (*n* = 6–10). ***F***, Quantification of time spent freezing after cadaverine exposure in 1 and 5 dph fish. ctrl versus cad *p* = 0.0081; 5 versus 5 dph cad *p* = 0.0074 (*n* = 5–8). ***G***, Quantification of odor avoidance index in controls, 1 dph, and 5 dph fish. Violin plots indicate mean, quartiles, and range. **p < 0.01; ***p < 0.001; ****p < 0.0001. One-way ANOVA with Tukey’s post hoc test.

Fish were exposed to hypoxic conditions in a hypoxic chamber formed by a sealed 12.7 cm depth × 8.9 cm height × 17.8 cm width glass container (Rubbermaid) outfitted with two gas ports. To displace dissolved oxygen (DO) in 300 ml of fish water, nitrogen gas (N_2_) was delivered through a submerged surgical tube connected to an N_2_ cylinder. A second outlet tube was positioned at the water surface to allow for excess gas exchange and maintain controlled circulation within the chamber. DO levels were continuously monitored with a submerged digital DO probe (RCYAGO, China; Fig. 1). Once the DO concentration reached 0.8 mg/L [a concentration shown to cause severe acute hypoxia in adult zebrafish (Yu and Li, 2011; Lee et al., 2018; Marino et al., 2020)]. nitrogen perfusion was stopped, and individual fish were placed in the sealed tank for 15 min. Following hypoxic exposure, fish were allowed to recover for 1 or 5 dph before being used for experiments. Control fish were subjected to identical handling procedures ensuring that any effects of handling stress, confinement, or fasting were equivalent across experimental groups.

### Olfactory-mediated behavioral assays

We used rectangular clear tanks (4.7 cm depth × 9.6 cm height × 15.8 cm width; AquaCulture) to assess behavioral responses to the odorant cadaverine. Each tank was filled with 1.5 L of fresh water from the zebrafish system in our facility. The tanks were fitted with surgical plastic tubing placed on the opposite side of the tank, slightly below the water level to avoid water disruption. Syringes were attached to the tubes and used for the simultaneous administration of 1 ml of odorant and PBS (vehicle) on opposite sides of the tank (Vorhees et al., 2025). Tubes and syringes used for cadaverine delivery were not used for PBS exposure to avoid cross-contamination. We used a digital camera to record fish swimming behaviors, placed behind a white panel that surrounded the tank to conceal the presence of investigators, in order to minimize fish distraction during testing. To assess the dispersal of the odorant solution, we injected 1 ml of a 100 µM cadaverine solution dyed with methylene blue in one side of the behavioral arena. We recorded the solution dispersal for 30 s and mapped the dye optical density (OD) using Adobe Photoshop.

Before experiments, fish were fasted in isolation for 48 h to standardize motivational state and minimize behavioral variability (Kermen et al., 2020; Masuda et al., 2024). On the day of the experiment, individual fish were placed in the experimental tank and acclimated for an hour total, with the final 30 min in the absence of experimenter-emitted sounds. During the acclimation and experimental periods, water within the experimental tank was kept still to ensure clear video recordings of each trial for analysis and to not disturb fish swimming patterns. Fish were recorded for 30 s before and 30 s after simultaneous exposure to odorant and PBS. The odorant solution consisted of 1 ml of 100 µM cadaverine (Sigma-Aldrich) in PBS, whereas the vehicle (control) consisted of PBS. Each fish underwent three to four trials on the same day. Following each trial, fish were placed in an alternate experimental tank with fresh water from the zebrafish facility to acclimate for 60 min before commencing the next trial. The side of the odorant delivery was alternated for each trial to control for potential side preferences or spatial bias. Water temperature in the behavioral arena decreased by 2.5°C ± 0.20 during the 1 h acclimation period, while ammonia levels and DO remained constant. Representative swimming traces were generated by manual tracing of video recordings to qualitatively facilitate visualization of fish positioning during trials.

### Scoring of olfactory-mediated behaviors

To analyze behavioral responses to cadaverine, we used recordings of the trials described above and manually coded behaviors in a blinded manner. The tracking software used is unable to assess the total time spent freezing or darting, which then necessitated manual coding of these behaviors (Calvo-Ochoa et al., 2025; Vorhees et al., 2025). The cumulative durations of darting, freezing, and avoidance behaviors were quantified (in seconds) during the preodor and odor-exposure periods. Two or three investigators independently coded recordings, and their scores were averaged. We first analyzed darting behaviors, defined as rapid, high-velocity escape–like movements characterized by abrupt acceleration and directional changes. We also assessed freezing, a behavior defined as the absence of body displacement for ≥1 s. To assess cadaverine-evoked spatial avoidance, the arena was divided into two equal halves to define odor and nonodor halves during analysis. To assess spatial avoidance, an odor avoidance index was calculated for both the prestimulus and poststimulus periods using the following formula: odor avoidance index = (*T* nonodor − *T* odor) / (total time). Prestimulus avoidance indices serve as a within-fish baseline to confirm the absence of inherent spatial preferences prior to odorant delivery and are shown alongside poststimulus values. Values near zero indicate no spatial preference while positive values indicate avoidance of the odorant side.

### Locomotor and exploratory assays in rectangular behavioral arena

We assessed the effects of hypoxia on locomotor ability and exploratory behaviors using the rectangular arena described above, to assess swimming parameters under identical conditions to the cadaverine olfactory assay. We fasted and acclimated individual fish as described above, recorded their behavior for 30 s in the absence of cadaverine, and then analyzed the swimming and exploratory parameters in the video recordings. The 30 s window was selected to match the duration of the cadaverine poststimulus recording period, allowing direct comparison of swimming parameters across identical conditions.

### Analysis of locomotion and exploratory parameters in rectangular behavioral arena

We first measured vertical displacement by dividing the arena into three equal vertical zones during analysis. We calculated the cumulative vertical distance traveled by counting the number of crosses across the three zones and multiplying them by zone height (in centimeter). To assess overall exploratory activity in the rectangular arena, the tank was divided into four equal quadrants. Exploratory activity was quantified as the total number of vertical and horizontal quadrant boundary crossings per trial.

### Locomotor and exploratory assays in behavioral circular arena

To comprehensively assess the effects of hypoxia on locomotion and exploratory behavior, we employed a second complementary assay to assess free-swimming parameters including speed, distance, and exploration rate without spatial constraints. For this, we used a circular arena, consisting of a 30-cm-diameter cylindrical behavioral tank filled with 2.5 L of water from the fish facility. Fish were fasted and acclimated in similar circular tanks before trials. We placed an overhead digital camera 1 m above the arena to record behavior for 30 s in the absence of cadaverine exposure and then analyzed swimming and exploratory parameters in the video recordings.

### Analysis of locomotion and exploratory parameters in circular behavioral arena

We used ToxTrac v024.1.1 (Rodriguez et al., 2018), an automated tracking software, to analyze behavior video recordings. We obtained the exploration rate from the software to quantify overall exploratory activity. Swimming distance (in centimeter) and speed (centimeter/second) were also obtained from ToxTrac.

### Tissue processing

Control fish and hypoxic fish after recovery were killed by over anesthetization with a solution of 0.03% tricaine (Sigma-Aldrich) at 4°C and then decapitated. Whole heads were fixed with 4% paraformaldehyde (PFA; Sigma-Aldrich) in PBS overnight (o/n) at 4°C. The next day, brains, olfactory organs, or whole heads were dissected and treated for IHC or histological staining.

Fixed brains and olfactory organs (formed by sensory olfactory epithelia) were washed and placed in 70% ethanol. The tissue was slowly dehydrated by incubations with increasing ethanol concentrations, followed by an incubation in xylene (Sigma-Aldrich). We embedded the dehydrated tissue by placing it in paraffin (Paraplast) and rapidly cooling to solidification o/n. Then, we obtained semiserial 10 µM sections of olfactory organs or brains in the coronal plane. These were adhered to charged slides (Thermo Fisher Scientific) and left to dry o/n at 37°C. For whole-head processing, after fixing in PFA, the tissue was decalcified using an RDO rapid decalcifying solution (Electron Microscopy Sciences) for 2 h at RT, washed with PBS, and placed in 70% ethanol. The tissue was dehydrated and embedded as described above, and then semiserial, 10 µm sections in the horizontal plane were obtained and adhered to charged slides.

### IHC

The mounted tissue was rehydrated by descending ethanol incubations and then subjected to antigen retrieval with a 10 mM sodium citrate (Sigma-Aldrich) solution, pH 6.0, at 70°C for 10 min. Next, slides were washed with PBS and blocked for at least an hour with a blocking buffer containing 3% normal goat serum (Vector Laboratories) and 0.4% Triton X-100 (Sigma-Aldrich). Following blocking, we incubated slides with primary antibodies (Table 1) o/n at RT and then incubated them with a fluorescently labeled secondary antibody (Table 1) with 1 µg/ml of 4′,6-diamidino-2-phenylindole (DAPI; BD Pharmingen) as a nuclear counterstain. Tissue sections were washed, coverslipped using PVA-DABCO (Sigma-Aldrich), and examined with a confocal laser-scanning microscope Nikon A1 and the NIS-Elements software.

**Table 1.** Antibodies used in the study

Antibody	Species	Dilution	Source	Catalog #
HuC/D (pan-neuronal marker)	Mouse	1:100	Thermo Fisher Scientific	A21271
GFAP (glial fibrillary acidic protein; astroglial marker)	Rabbit	1:500	DAKO	Z033429-2
Lcp1 (lymphocyte cytosolic protein 1; leukocyte marker)	Rabbit	1:100	Genetex	GTX124420
PCNA (proliferating cell nuclear antigen; marker for cell proliferation)	Mouse	1:1,000	Sigma-Aldrich	P8825
Fluorescently labeled secondaries: anti -mouse and anti-rabbit, IgG	Goat	1:200	Invitrogen	A11001 A11005 A11008 A11012

### Histological stainings and goblet cell quantification

Sectioned OE samples were deparaffinized with xylene, rehydrated by descending ethanol incubations, and stained with Alcian Blue and nuclear fast red (Vector Laboratories). Then, we dehydrated the samples and mounted them using Permount (Thermo Fisher Scientific). The stained tissue was examined under light microscopy with Leica DM5000 B Automated Upright Microscope and analyzed with the Application Suite X System software (Leica).

We identified goblet cells by their staining with Alcian blue, rounded morphology, and location within the olfactory lamellae. We manually quantified the number of glands per single olfactory lamellae in images taken at 40× magnification. We counted and averaged 4–6 sections per animal. To measure olfactory lamellae thickness and goblet cell diameter (in micrometer) in the stained tissue, we used the Application Suite X System software (Leica).

### Cell quantification and densitometry

We used Adobe Photoshop (Adobe) to quantify cells and to determine the relative fluorescent intensity value of the immunostained tissue in images taken at 20× magnification. We quantified and averaged 3–5 tissue sections per animal. For cell quantification, we manually counted PCNA^+^ somata in OE and coronal telencephalon sections.

Fluorescence signal from immunostainings against Lcp1 and GFAP in OE and OB tissue, respectively, was quantified using luminosity measurements obtained from grayscale images obtained from the confocal microscope. We measured mean luminosity values for each region of interest and a background region lacking specific signal. A relative fluorescence intensity value was calculated as relative fluorescence intensity = −log (mean luminosity of background / mean luminosity of region of interest), providing a background-normalized measure of immunoreactivity in arbitrary units (a.u.). Images were acquired using identical settings for all samples.

### TTC reduction assay

We evaluated brain mitochondrial dehydrogenase activity by using a 2,3,5-triphenyltetrazolium chloride (TTC) reduction assay. Immediately following fish decapitation, we dissected whole brains and incubated them in a 2% TTC (Sigma-Aldrich) solution in PBS at 37°C for 40 min, shielded from light. Then, TTC was removed, and 10% PFA was added to terminate the reaction. After 10 min, brains were washed in PBS, fixed in 4% PFA, and kept at 4°C o/n. The next day, we imaged the dorsal and ventral sides of TTC-stained brains using an Olympus SZ61 stereo microscope with a digital EP50 camera (Olympus) and then converted the images to 8 bit gray scale using Photoshop (Adobe). We obtained the mean luminosity values for the dorsal and ventral sides of the OB and the rest of the brain. Luminosity values were converted to OD by the following formula: OD = −log (intensity of background / intensity of area of interest; Braga et al., 2013; Braga et al., 2016). We calculated the percent difference relative to controls using the following formula: percent difference = (OD sample − mean OD control) / mean OD control value × 100. Percent difference values were calculated relative to control and multiplied by −1 so that negative values (more luminosity due to less TTC staining) indicate reduced mitochondrial activity.

### Statistical analysis

Comparisons between groups were carried out using analysis of variance (ANOVA) with Tukey post hoc tests or unpaired *t* tests. *P* values <0.05 were considered significant. Effect sizes for all one-way ANOVAs are reported as Cohen's *f*, calculated from eta-squared values derived from the ANOVA sum of squares as *f* = √(η^2^/(1 − η^2^)). We used GraphPad Prism 10 software for all statistical analyses and graphs (GraphPad).

## Results

### Acute hypoxic exposure transiently impairs olfactory function

We sought to investigate the effects of acute hypoxic exposure on olfactory function. For this, fish were subjected to severe hypoxia in a hypoxic chamber (Fig. 1*A*) and allowed to recover for 1 or 5 dph) assess recovery. Controls were maintained at standard DO concentrations. First, we assessed whether this hypoxic paradigm led to compromised olfactory function by analyzing olfactory-mediated responses to cadaverine, a colorless diamine released by decaying flesh that elicits robust and stereotypical aversive responses in zebrafish (Hussain et al., 2013). Single fish were acclimated to a rectangular tank and exposed to cadaverine for testing (Fig. 1*B*). We recorded swimming behaviors 30 s before and 30 s after odorant exposure and quantified three aversive behaviors: darting, freezing, and odor-evoked avoidance. To assess the latter, we calculated an odor avoidance index for both the pre- and postcadaverine periods by measuring the cumulative time spent in the odor and nonodor halves of the arena. Importantly, the cadaverine solution remains in the odor half of the arena for the duration of the trial (Fig. 1*C*,*D*, highlighted in pink). Representative swimming trajectories are shown in Figure 1*D*. Control fish displayed expected aversive behavioral responses to cadaverine, including darting (Fig. 1*E*; *p* = 0.0001; Cohen's *f* = 1.25) and freezing (Fig. 1*F*; *p* = 0.0081; Cohen's *f* = 0.87). In the avoidance assay, preodor avoidance indices clustered around zero in all groups (ctrl, 1 dph, and 5 dph), confirming the absence of inherent spatial preferences prior to odorant delivery (Fig. 1*G*). Following cadaverine exposure, control fish showed a significant increase in avoidance index compared with their preodor baseline (Fig. 1*G*; *p* = 0.0058; Cohen's *f* = 1.23). In the 1 dph group, all olfactory-mediated responses to cadaverine were impaired: darting (Fig. 1*E*), freezing (Fig. 1*F*), and odor avoidance (Fig. 1*G*), indicated by no significant changes from their baseline preodor values. These results indicate a broad impaired olfactory response to cadaverine. Remarkably, by 5 d, fish regained olfactory sensitivity to cadaverine, demonstrated by restored darting (5 dph vs 5 dph cad *p* = 0.0012; Cohen's *f* = 1.25; Fig. 1*E*), freezing (5 vs 5 dph cad *p* = 0.0074; Cohen's *f* = 0.87; Fig. 1*F*), and avoidance behaviors (*p* = 0.0244; Cohen's *f* = 1.23; Fig. 1*G*).

### Gross locomotion and spatial exploration are not affected by hypoxic treatment

**Figure 2.**
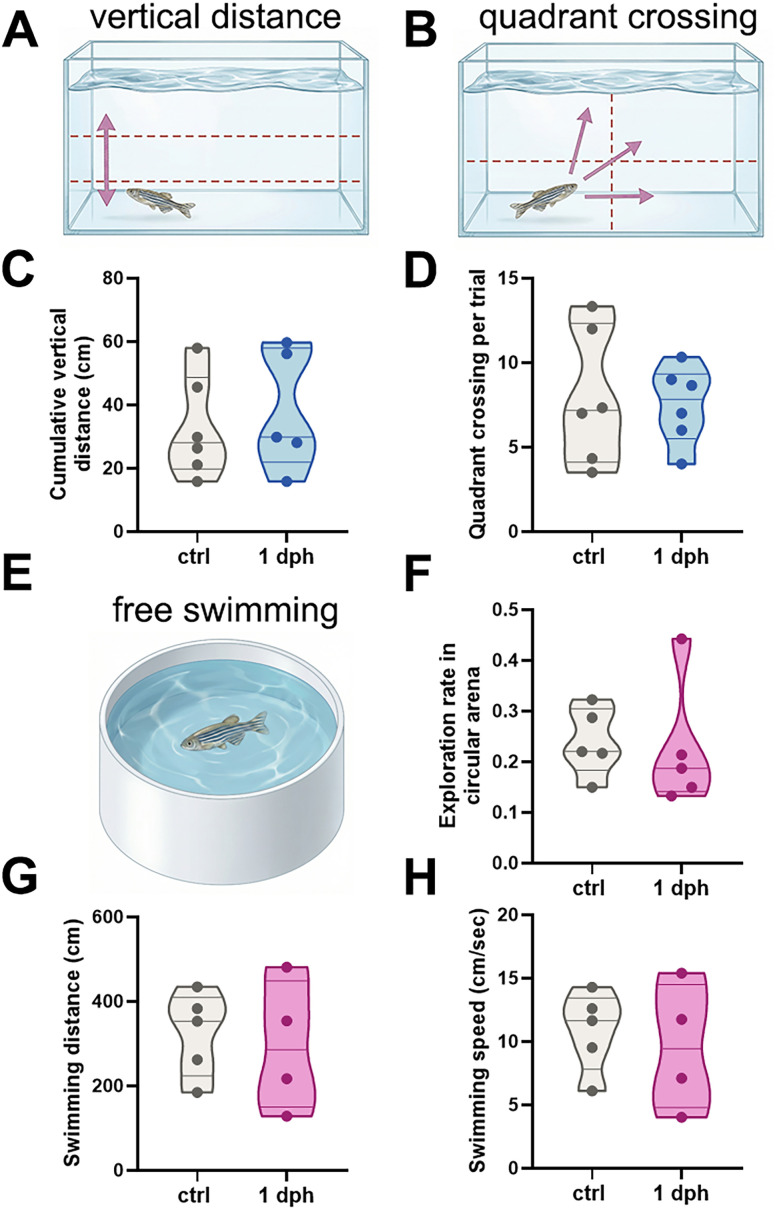
Exploratory behaviors and swimming parameters in control and hypoxic fish. ***A***, Diagram of the behavioral tank depicting three vertical zones used to quantify vertical distance displacement. ***B***, Behavioral tank schematic depicting four zones used to quantify zebrafish quadrant boundary crossings. ***C***, Quantification of cumulative vertical distance in controls and 1 dph fish (*n* = 6). ***D***, Quantification of quadrant crossing per trial in controls and 1 dph fish (*n* = 6). ***E***, Schematic of the behavioral circular arena used for free-swimming assessment. ***F***, Quantification of exploration in a circular arena in controls and 1 dph fish (*n* = 5). ***G***, Quantification of swimming distance in a circular arena in controls and 1 dph fish (*n* = 5). ***H***, Quantification of swimming speed in a circular arena in controls and 1 dph fish (*n* = 5). Violin plots indicate mean, quartiles, and range. Unpaired *t* test.

To confirm that the hypoxia-mediated olfactory behavioral deficits observed were not attributable to locomotor impairment caused by hypoxia, we assessed gross locomotion parameters and exploratory behaviors. To quantify vertical and whole-tank exploration, the tank was virtually divided into thirds and quadrants during video analysis, allowing us to assess exploration across the arena (Fig. 2*A*,*B*). We found no significant differences in cumulative vertical distance or number of quadrants crossing between groups (Fig. 2*C*,*D*), suggesting that exploratory behaviors are largely unaffected by hypoxia exposure. Then, we assessed whether hypoxia impairs gross locomotion. For this, individual fish were allowed to free-swim in a circular, larger arena without spatial constraints (Fig. 2*E*). We calculated the exploration rate and quantified the swimming speed and distance and found no significant differences across groups (Fig. 2*F*–*H*).

### Acute hypoxia causes transient neurodegeneration of the OE and alterations in nasal mucus

**Figure 3.**
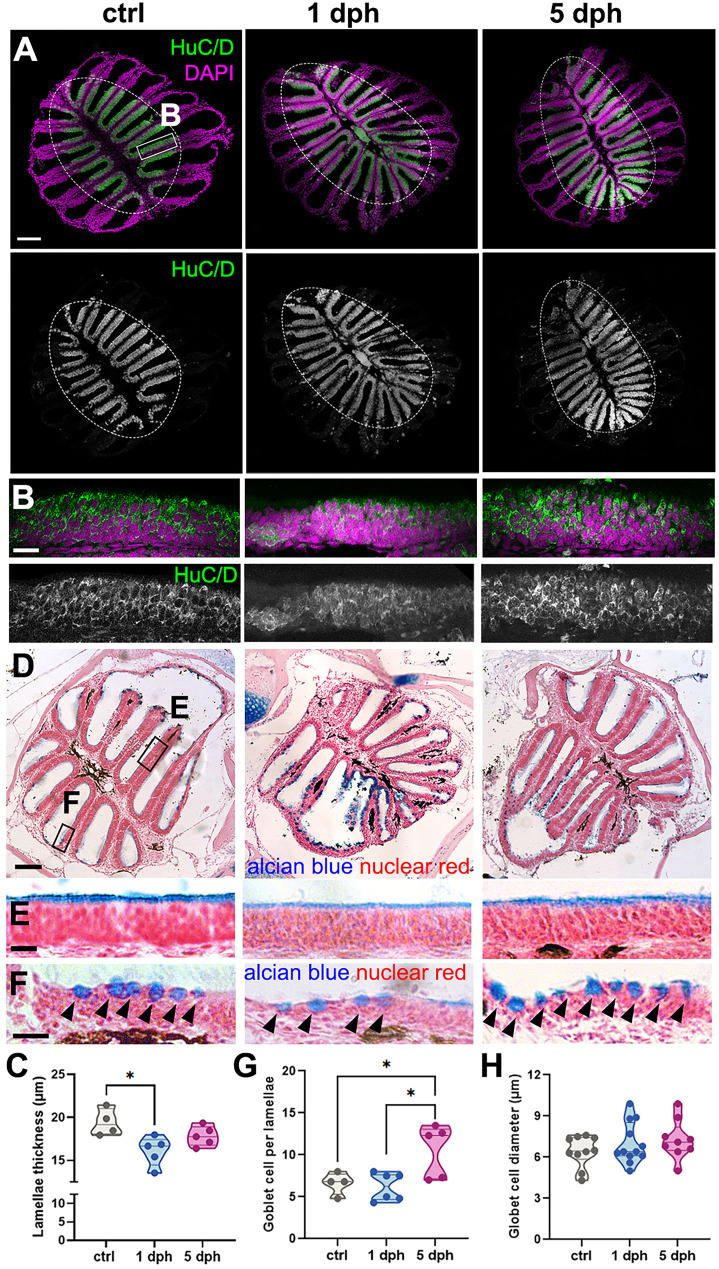
Effects of acute hypoxic exposure on OE structural integrity and the olfactory mucus layer. ***A***, Immunohistochemical stainings of HuC/D in OE sections from controls, 1 dph, and 5 dph fish. ***B***, Magnified views of the sensory epithelium (HuC/D^+^) of ***A***. ***C***, Quantification of olfactory lamellae thickness in controls, 1 dph, and 5 dph groups (*n* = 4–5). ctrl versus 1 dph *p* = 0.0162 (*n* = 4–5). ***D***, Alcian blue and nuclear fast red histological stainings of OE sections of controls, 1 dph, and 5 dph fish. ***E***, Magnified views of the sensory epithelium of ***D***. The epithelial mucus layer is evident in blue staining. ***F***, Magnified views of the nonsensory epithelium of ***D***. Goblet cells are indicated by black arrowheads. ***G***, Number of goblet cells per olfactory lamellae in controls, 1 dph, and 5 dph groups. ctrl versus 5 dph. *p* = 0.0498; 1 versus 5 dph *p* = 0.0170 (*n* = 4–6). ***H***, Quantification of goblet cell diameter (in micrometer) of controls, 1 dph and 5 dph fish (*n* = 9–12). Green, HuC/D; magenta, DAPI. Scale bar, 100 µm in ***A***; 20 µm in ***B***, ***C***, and ***D***. Violin plots indicate mean, quartiles, and range. *p < 0.05. One-way ANOVA with Tukey’s post hoc test.

To determine whether acute hypoxia causes OSN loss and epithelial thinning that could be associated with the olfactory dysfunction observed, we examined the structural integrity of the sensory OE by using IHC against HuC/D (pan-neuronal marker). Our results revealed striking changes in the OE of hypoxic fish. We observed a significant thinning of the olfactory lamellae (Fig. 3*A*–*C*; *p* = 0.0162; Cohen's *f* = 1.02) along with a noticeable disruption of the HuC/D^+^ sensory layer of the epithelium (Fig. 3*B*), indicative of OSN loss. Echoing the recovery of olfactory-mediated behaviors by 5 dph, OSNs in the sensory epithelium regained their density and control morphology at this timepoint (Fig. 3*A*–*C*). Next, we qualitatively assessed the integrity of the mucus layer—through which odorants must dissolve before binding to OSN receptors—by performing Alcian blue histological stainings, which labels acidic mucopolysaccharides and identifies mucus-producing goblet cells. We found that the mucus layer within the sensory epithelium appeared thinner and disorganized in 1 dph fish when compared with controls (Fig. 3*D*,*E*). In these magnifications of the histological staining, the significant thinning of the neuroepithelium (Fig. 1*B*,*C*) is also evident. Moreover, by 5 d, the mucus layer had returned to control appearance along with recovery of the epithelium (Fig. 3*D*,*E*). Furthermore, we examined mucus-producing goblet cells, identifiable by their characteristic rounded shape and strong Alcian blue positivity (Fig. 3*D*,*F*). Interestingly, although we did not observe changes in goblet cell numbers in 1 dph fish, we found elevated numbers at 5 dph compared with the other groups (Fig. 3*F*,*G*; ctrl vs 5 dph; *p* = 0.0498; 1 vs 5 dph *p* = 0.0170; Cohen's *f* = 1.01). Goblet cell diameter remained unchanged across experimental groups (Fig. 3*F*,*G*).

### Peripheral leukocytic infiltration to the OE follows hypoxic exposure

**Figure 4.**
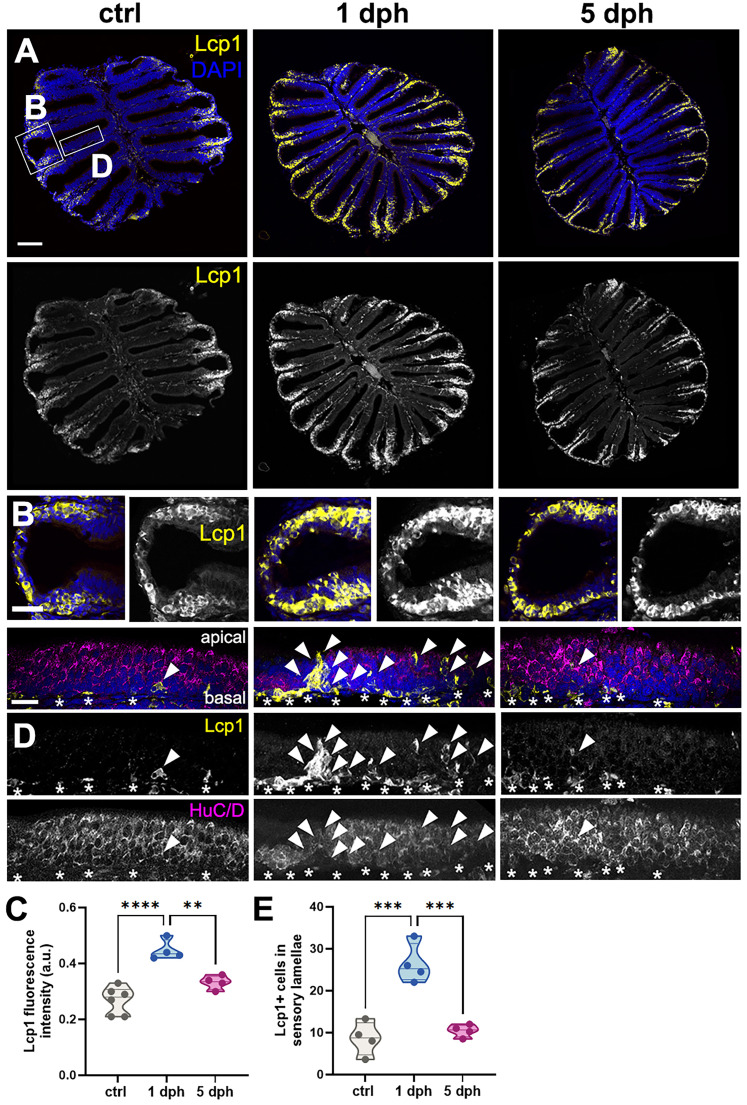
Effects of hypoxia on leukocytic recruiting to the OE. ***A***, Immunohistochemical staining of Lcp1 in OE sections from controls, 1 dph, and 5 dph fish. ***B***, Magnified views of the outer nonsensory epithelial fold of ***A***. ***C***, Quantification of Lcp1 relative fluorescent intensity in sections from ***A***. ctrl versus 1 dph *p* < 0.0001; 1 versus 5 dph *p* = 0.0051 (*n* = 4–6). ***D***, Magnified views of the sensory epithelium of OE sections double immunohistochemically stained against HuC/D and Lcp1. Lcp1^+^ cells present in the basal membrane are indicated by asterisks, while white arrowheads indicate Lcp1^+^ leukocytes infiltrating apical sensory layers. ***E***, Quantification of Lcp1^+^ cells found in the sensory lamellae of ctrl, 1 dph, and 5 dph groups. ctrl versus 1 dph *p* = 0.0002; 1 versus 5 dph *p* = 0.0005 (*n* = 4). Yellow, Lcp1; magenta, HuC/D. Scale bar, 100 µm in ***A***, 20 µm in ***B***, and 40 µm in ***D***. Violin plots indicate mean, quartiles, and range. **p < 0.01, ***p < 0.001, ****p < 0.0001. One-way ANOVA with Tukey post hoc test.

Given the well-documented role of leukocytic infiltration in peripheral neural degeneration in zebrafish (Villegas et al., 2012; Palominos et al., 2022), we examined the posthypoxic inflammatory response in the OE by quantifying Lcp1 (pan-leukocytic marker) immunoreactivity. These experiments revealed a significant increase in Lcp1 signal in the OE lamellae of 1 dph fish when compared with controls, with signal returning to control levels in 5 dph groups (Fig. 4*A*–*D*; ctrl vs 1 dph *p* < 0.0001; 1 vs 5 dph *p* = 0.0051; Cohen's *f* = 2.08). Upon further examination, we noticed an increase in the number of Lcp1^+^ cells in the sensory lamellae. To confirm this, we performed a double IHC staining against HuC/D and Lcp1 and found an increased number of individual Lcp1^+^ cells found within the HuC/D^+^ sensory epithelium in the 1 dph group (Fig. 4*D*,*E*; *p* = 0.0002; Cohen's *f* = 2.51). Notably, in control fish, most leukocytes are found underlying the basal lamina propria of the epithelium (Fig. 4*D*, indicated by asterisks), while in the 1 dph group, an increased number of Lcp1^+^ cells were observed infiltrating apical layers, surrounding OSNs (Fig. 4*D*, indicated by white arrowheads). We also noticed changes in leukocyte morphology in the 1 dph group: resting leukocytes in control fish were small and rounded, whereas activated leukocytes in hypoxic fish adopted an enlarged and/or ameboid phenotype (Fig. 4*B*,*D*). Conversely, the inflammatory response subsided within 5 d, as indicated by the Lcp1^+^ presence in the sensory lamellae returning to control levels in the 5 dph group (Fig. 4*D*,*E*; 1 vs 5 dph *p* = 0.0005; Cohen's *f* = 2.51).

### Acute hypoxia induces central reactive astrogliosis and mitochondrial dysfunction in the OB

**Figure 5.**
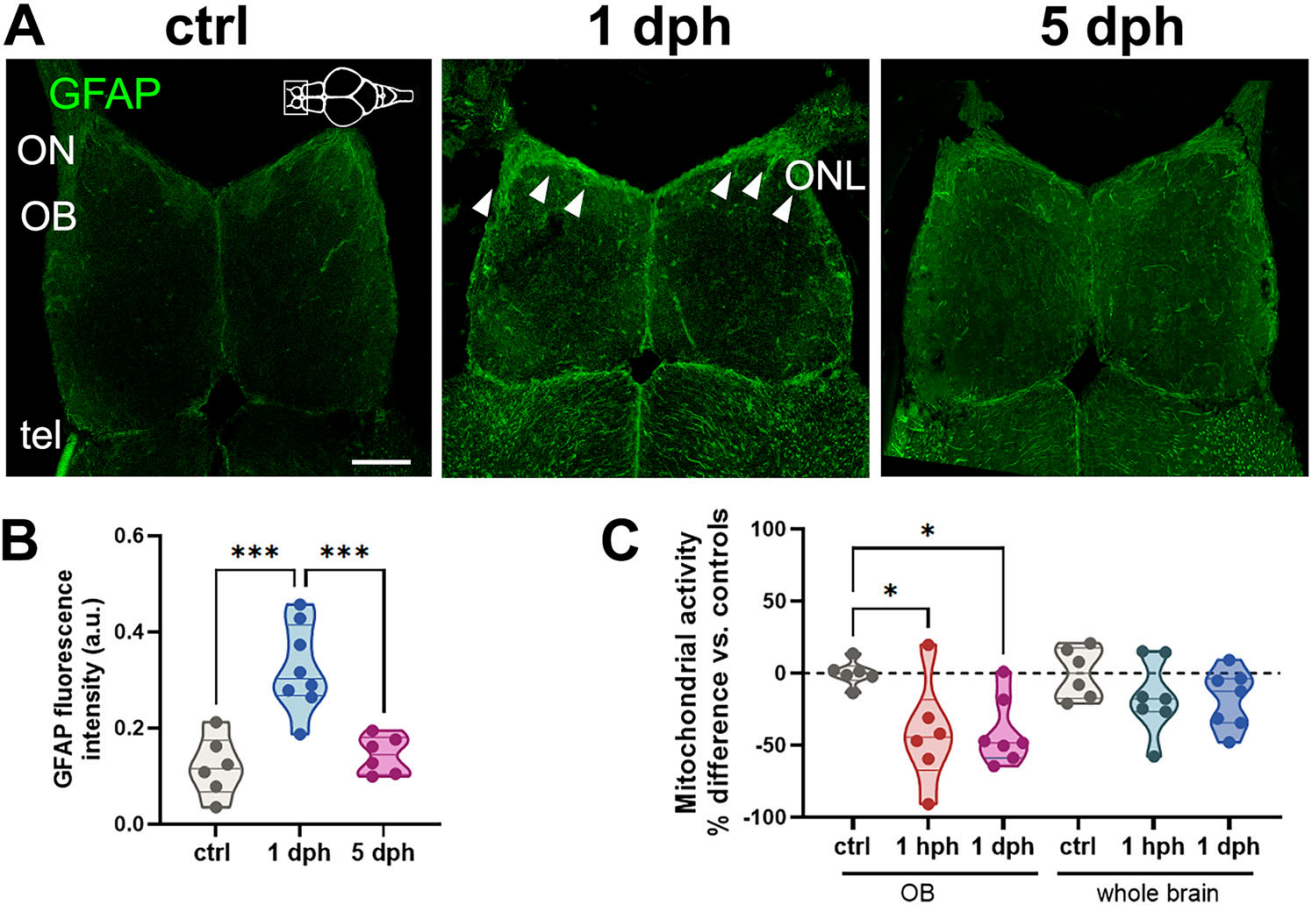
Effects of hypoxia on reactive astrogliosis and mitochondrial activity in the OB. ***A***, Glial fibrillary acidic protein (GFAP) immunohistochemical stainings of horizontal sections of the OB from controls, 1 dph, and 5 dph fish. Increased GFAP staining along the ON and the ONL in the OB is indicated with white arrowheads. ***B***, Quantification of GFAP relative fluorescent intensity in OB sections from (***A***). ctrl versus 1 dph *p* = 0.0001; 1 versus 5 dph *p* = 0.0005 (*n* = 6–8). ***C***, Quantification of mitochondrial activity percent difference relative to controls in the OB and whole brains of ctrl, 1 hph, and 1 dph groups. OB, ctrl versus 1 hph *p* = 0.0442; ctrl versus 1 dph *p* = 0.0393 (*n* = 6–7). Green, GFAP. Scale bar, 100 µm. Violin plots indicate mean, quartiles, and range. *p < 0.05; ***p < 0.001. One-way ANOVA with Tukey’s post hoc test.

Due to the selective vulnerability of the OB to metabolic dysfunction and absence of OE afferents, we investigated the responses of astroglia in the OB and OECs in the ON using IHCs against GFAP. We observed a significant increase in GFAP immunoreactivity in the OB at 1 dph compared with normoxic controls, particularly along the ON layer (ONL; Fig. 5*A*,*B*; *p* = 0.0001; Cohen's *f* = 1.46), indicative of robust astroglial activation following hypoxic exposure. By 5 dph, GFAP immunoreactivity returned to control levels, suggesting resolution of the astroglial response in parallel with behavioral recovery (Fig. 5*A*,*B*; 1 vs 5 dph *p* = 0.0005; Cohen's *f* = 1.46).

Given that hypoxia compromises mitochondrial function, we quantified mitochondrial dehydrogenase activity in the OB using a TTC colorimetric assay validated in zebrafish brains (Yu and Li, 2011). Mitochondrial activity could be affected rapidly by hypoxia, thus we quantified TTC staining after 1 h (1 hph) and 1 d post hypoxic exposure (1 dph) by measuring the OD of the OB and the rest of the brain. We then converted these values to percent difference relative to controls in mitochondrial activity. OB from 1 hph and 1 dph fish displayed larger unstained areas and significantly higher luminosity values compared with controls, indicating a marked reduction in mitochondrial activity following hypoxic exposure (Fig. 5*C*; ctrl vs 1 hph *p* = 0.0442; ctrl vs 1 dph *p* = 0.0393; Cohen's *f* = 0.73). Interestingly, mitochondrial activity levels of the rest of the brain of hypoxia-exposed fish did not differ from controls (Fig. 5*C*).

### Hypoxic exposure induces cell proliferation within the OE and telencephalic ventricular zone

**Figure 6.**
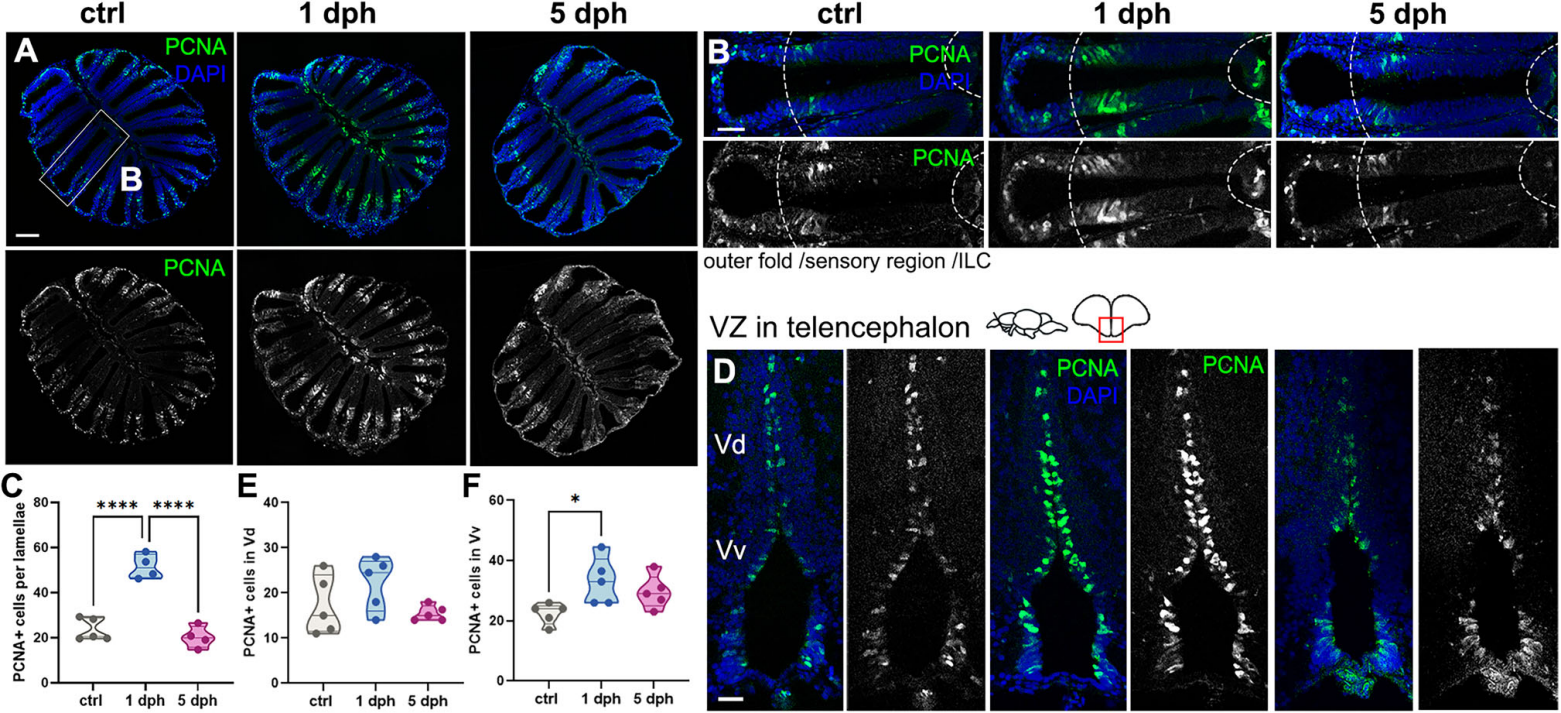
Effects of acute hypoxic exposure on cell proliferation in the OE and telencephalon. ***A***, Proliferating cellular nuclear antigen **(**PCNA) immunohistochemical stainings of OE sections from controls, 1 dph, and 5 dph fish. ***B***, Magnified views from ***A***. Dashed lines indicate the following anatomical divisions of the lamellae: outer nonsensory epithelial fold (left), sensory region (middle) and ILC (right). ***C***, Quantification of PCNA^+^ cells in single olfactory lamellae from ***A***. ctrl versus 1 dph *p* < 0.0001; 1 versus 5 dph *p* < 0.0001(*n* = 4–5). ***D***, PCNA immunohistochemical stainings of coronal telencephalon sections from controls, 1 dph, and 5 dph groups. The Vd and Vv regions within the ventricular zone (VZ) are indicated. ***E***, Quantification of PCNA^+^ cells within the Vd telencephalic region shown in ***C*** (*n* = 5). ***F***, Quantification of PCNA^+^ cells within the Vv telencephalic region from ***C***. ctrl versus 1 dph *p* = 0.0329 (*n* = 5). Violin plots indicate mean, quartiles, and range. Green, PCNA; blue, DAPI. Scale bar, 100 µm in ***A*** and ***D***; 20 µm in ***B***. Violin plots indicate mean, quartiles, and range. *p < 0.05; ****p < 0.0001. One-way ANOVA with Tukey post hoc test.

We sought to determine whether the structural and functional recovery observed at 5 dph is supported by active cell proliferation. For this, we used PCNA IHC to assess proliferating cells in the OE and the ventroventral (Vv) and ventrodorsal (Vd) regions of the telencephalic ventricular zone—a well-characterized neurogenic niche posterior to the OB that drives regeneration and repair following neural injury (Adolf et al., 2006; März et al., 2011). Control fish exhibited constitutive cell proliferation in the OE, in particular in the outer epithelial fold (Fig. 6*A*,*B*). Our results indicate that hypoxia induces cell proliferation within the OE, as evidenced by a significant increase in PCNA^+^ cells at 1 dph when compared with controls (Fig. 6*A*–*C*; *p* < 0.0001; Cohen's *f* = 3.10). Interestingly, PCNA immunoreactivity appeared increased within the sensory region and the interlamellar curve (ILC) in the 1 dph group (Fig. 6*B*, indicated by dashed lines). Moreover, PCNA^+^ numbers returned to control levels by 5 dph (Fig. 6*A*–*C*; 1 vs 5 dph *p* < 0.0001; Cohen's *f* = 3.10). Similarly, we noticed a significant increase in cell proliferation within the ventral telencephalon (Vv) of 1 dph fish when compared with controls (Fig. 6*D*,*F*; *p* = 0.0329; Cohen's *f* = 0.85). However, we found no difference in PCNA^+^ profiles in the Vv between the two hypoxic groups (Fig. 6*D*,*E*), indicating that proliferation in the ventral telencephalon remains sustained at 5 dph. Additionally, no differences were found in the Vd region across groups (Fig. 6*D*,*F*).

## Discussion

In this study, we investigated the functional and structural effects of acute oxygen deprivation on the olfactory system. Adult zebrafish is an advantageous model organism for studying hypoxia-induced repair responses because they are able to withstand low-oxygen conditions and exhibit robust widespread regeneration.

### Acute hypoxia transiently impairs olfactory-mediated behavior without affecting gross locomotion

We show that acute hypoxia causes olfactory dysfunction, as demonstrated by reduced responses to cadaverine at 1 dph. Cadaverine is produced during tissue decomposition and activates TAAR13c-expressing OSNs, eliciting strong odorant-evoked aversive responses (Hussain et al., 2013). Notably, olfactory-mediated responses to cadaverine were fully restored at 5 dph, indicating that olfactory dysfunction is reversible. Importantly, our hypoxic paradigm does not impair swimming or exploratory behaviors, indicating that the impaired responses to cadaverine arise due to olfactory loss rather than locomotor impairment. This is consistent with prior reports showing that, while motor deficits are observed at very early recovery timepoints (between 1 and 2 hph), locomotion normalizes as soon as 3 hph (Braga et al., 2013; Lee et al., 2018; Marino et al., 2020). This temporal pattern suggests that hypoxia-mediated motor deficits may reflect transient neuromuscular metabolic stress rather than impairment of neuronal motor circuits (Cadiz et al., 2019). Normal swimming resume as compensatory responses such as improved oxygen usage are deployed (Van Ginneken et al., 1995; Esbaugh et al., 2009).

Our findings align with reports of posthypoxia olfactory decline across different species (Bigdaj et al., 2018; Kim et al., 2023). Mice exposed to chronic intermittent hypoxia exhibited broad olfactory alterations (Hernández-Soto et al., 2021). Similarly, humans exposed to hypoxic conditions due to sleep apnea or experimental high altitude experience olfactory dysfunction, as evidenced by a lowered olfactory threshold (Huppertz et al., 2018; Binar and Gokgoz, 2021). Our study extends these findings by providing a characterization of posthypoxia cellular and morphological changes across the olfactory axis.

This work focused on studying olfactory-evoked responses to cadaverine, but whether hypoxia impairs olfactory responses to other odorant categories remains to be explored. Notably, moderate hypoxia reduces olfactory sensitivity to amino acids in the teleost gilt-head seabream (*Sparus aurata*; Tigert et al., 2025), suggesting that hypoxia could lead to reduced food-seeking behaviors. The cellular and morphological mechanisms underlying this transient olfactory dysfunction are discussed in the following sections.

### Peripheral neurodegeneration and mucus disruption impair olfactory function

The structural integrity of the olfactory organ is crucial for olfactory signal transduction. Our findings revealed a significant thinning of the sensory OE lamellae at 1 dph, indicative of OSN loss and consistent with the deficits in olfactory function observed at this timepoint. These structural alterations recovered by 5 dph, paralleling the restoration of olfactory-mediated behaviors. Our findings confirm results from a study in mice that described a posthypoxia reduced expression of olfactory marker genes in the OE (Kim et al., 2023).

The OE is particularly susceptible to low oxygen because it is a neuron-rich tissue that relies heavily on diffusion from the external environment rather than a direct blood supply (Bigdai et al., 2019). Olfactory signal transduction is an energetically demanding process that relies on robust ATP production in the dendritic knob and processing of extracellular glucose in odorant cilia (Villar et al., 2017). Thus, a reduction in available oxygen in OSNs could be expected to impair oxidative phosphorylation and reduce ATP production, thereby diminishing olfactory sensitivity. This is consistent with a report showing that hypoxia reduces amino acid-evoked OSN potentials suggesting that the initial stages of odorant recognition are impaired by low oxygen, possibly through ATP reduction (Tigert et al., 2025). A limitation of our study is that we did not assess mitochondrial activity in the OE, because olfactory organs pigment can confound the results of colorimetric TTC assays.

In addition to OSN degeneration, we propose that the disruption of the nasal mucus layer might a contributing factor on the olfactory dysfunction observed. The mucus layer serves two important functions: it acts as a medium for the diffusion of both odorants and oxygen. Odorants must dissolve in nasal mucus before accessing olfactory receptors within OSN cilia (Rygg et al., 2013), while DO from the water diffuses to the mucus layer to the underlying neuroepithelium (Cox, 2008); thus the thickness and composition of nasal mucus directly affect the rate of diffusion of these important solutes. Moreover, sustentacular cells in the OE release glucose to the mucus layer where olfactory cilia incorporate it as energy source (Nunez-Parra et al., 2011). Thus, disruption of the nasal mucus could further impair OSN metabolic activity. Therefore, we propose that hypoxia-induced disruption of the mucus layer may both impair odorant transduction and further exacerbate the oxygen deficit experienced by OSNs.

While we were unable to determine the thickness of the mucus layer with our current techniques, our qualitative examination revealed histological changes within this layer as supported by our findings on goblet cell number. Interestingly, we observed an increase in the number of goblet cells at a timepoint when the OE's morphology had already recovered. This delayed goblet cell expansion suggests that mucosal remodeling continues beyond the window of hypoxic insult, possibly reflecting a compensatory response to the epithelial damage. Notably, while research on the impact of low oxygen in olfactory mucus-producing glands is scarce, prolonged hypoxia has been associated with increased mucus production and rearrangement of mucus-secreting cells in other epithelial tissues (Polosukhin et al., 2011), consistent with our findings, which contribute to this underexplored area of olfactory physiology.

### Transient inflammation in the OE coincides with functional and structural recovery

The robust leukocytic response in the olfactory organ following hypoxic exposure reflects the dynamic role that immune cell recruitment plays in response to injury in the peripheral nervous system (PNS) in zebrafish (Villegas et al., 2012; Palominos et al., 2022). Neutrophils are the most abundant type of circulating leukocytes and are typically the first immune cells recruited to sites of tissue injury (Harvie and Huttenlocher, 2015; Mokhtar et al., 2023), while macrophages are subsequently recruited to aid in debris removal, which facilitates tissue repair (Carrillo et al., 2016; Var and Byrd-Jacobs, 2020; Calvo-Ochoa et al., 2025).

The increase in Lcp1^+^ density following hypoxia, the infiltration into the neuroepithelium, and changes in cell morphology indicate leukocytic recruitment, consistent with prior reports of neutrophils and macrophages migration to the injured OE (Palominos et al., 2022; Calvo-Ochoa et al., 2025). A potential mechanism by which hypoxia might promote leukocytic migration to the OE is through the hypoxia-inducible factor signaling. This pathway leads to upregulation of genes that promote leukocyte recruitment, survival, and immune activity (Walmsley et al., 2005; He et al., 2022).

While inflammation is essential for initiating tissue repair, its resolution is required to enable regenerative processes. Our findings show that the inflammatory response subsides by 5 d, concurrent with OE's morphological recovery. The transient nature of the leukocytic response is consistent with evidence of acute inflammation as a key regulator of neuronal repair and regeneration in zebrafish (Var and Byrd-Jacobs, 2020). Acute inflammation has been shown to enhance proliferation of neural progenitors and subsequent neurogenesis following injury in different regions of the zebrafish brain, spinal cord, and PNS (Kyritsis et al., 2012; Villegas et al., 2012; Carrillo et al., 2016; Tsarouchas et al., 2018). On the other hand, our understanding of the role of leukocytes in the OE remains limited. In mice, OE-infiltrating neutrophils express neurogenesis-related genes following damage (Ogawa et al., 2021), while attenuation of inflammatory processes in the injured OE impaired cell proliferation (Chen et al., 2017). This raises the possibility that the leukocytic response observed might support repair and regeneration of lost OSNs. While leukocytic infiltration reflects the peripheral inflammatory response to hypoxia, concurrent changes in the OB suggest that the injury extends beyond the epithelium, affecting central olfactory processing, as described below.

### Early mitochondrial dysfunction and astrogliosis reveal inherent susceptibility of the OB

Our findings show that posthypoxia olfactory dysfunction is associated with structural and metabolic alterations along the olfactory axis. Our TTC data reveal that mitochondrial dehydrogenase activity in the OB is reduced as early as 1 h and that it continues to be reduced throughout the first day, while mitochondrial activity in the rest of the brain remains unchanged. This suggests that the OB has an inherent susceptibility to hypoxic insult. Other reports have shown that severe hypoxic exposure decreases mitochondrial dehydrogenase activity in the telencephalon and optic tectum, with mitochondrial activity remaining reduced for up to 24 h before normalizing by 48 h posthypoxia (Braga et al., 2013; Braga et al., 2016). Furthermore, repeated hypoxic exposure triggers downregulation of brain glucose levels and several glucose metabolites, with supplementation of glucose and glucosamine restoring learning and memory impairment (Park et al., 2021). Collectively, these results demonstrate that low oxygen widely impairs oxidative phosphorylation and glycolytic processes in the zebrafish brain. Our work extends these findings by demonstrating OB's inherent vulnerability to low oxygen, pointing to differences in metabolic demands and/or redox protective mechanisms across brain regions.

Hypoxia suppresses mitochondrial oxidative phosphorylation, reducing ATP production and increasing AMP levels, which collectively impair energy-demanding neuronal processes (Douglas et al., 2010; Fuhrmann and Brüne, 2017; Dhillon and Richards, 2018; Allen et al., 2020). In the context of the OB, where odorant-evoked synaptic transmission in the glomerular layer is particularly oxygen-consuming (Nawroth et al., 2007; Lecoq et al., 2009), this metabolic disruption could impair olfactory signal processing, amplifying the peripheral deficit caused by OSN degeneration.

Concurrent with mitochondrial dysfunction, we observed an increase in GFAP immunoreactivity particularly along the ONL, with resolution by 5 dph. GFAP is expressed by multiple glial populations in the OB, including astroglia and OECs, the latter being particularly expressed in the ONL where we observed the greatest increase in signal at 1 dph (Lazzari et al., 2014). OECs are specialized glia that envelope OSN axons as they project from the OE to the OB and are known to support olfactory neurogenesis and axonal regeneration (Franklin and Barnett, 1997; Chuah and West, 2002; Raisman and Li, 2007). Therefore, our observations of GFAP signal in the ON may reflect activation of OECs in addition to classical reactive astrogliosis.

Importantly, astroglia can exert both neuroprotective and neuroinflammatory effects in response to hypoxia, releasing neurotrophic factors and antioxidants that protect neurons from hypoxic injury (Marina et al., 2016), while also producing proinflammatory cytokines and reactive oxygen species that may exacerbate damage (Merelli et al., 2021). The resolution of GFAP immunoreactivity by 5 d, coinciding with functional recovery, suggests that in our model the astroglial response is ultimately protective, consistent with other injury models in which astrogliosis has been reported as regenerative (Pellegrini et al., 2007; Kroehne et al., 2011; Baumgart et al., 2012; Skaggs et al., 2014).

### Compensatory cell proliferation underlies rapid recovery

The remarkable and rapid recovery of olfactory function by 5 dph—despite the severity of the peripheral and central disruption observed at 1 dph—points to extensive compensatory mechanisms actively driving structural repair across the olfactory axis. The robust increase in cell proliferation throughout the OE supports the hypothesis that neurogenic mechanisms occur after hypoxic insult to replace damaged or lost OSNs. This damage-responsive proliferative response is consistent with the well-documented abilities of progenitor cell populations to drive neuronal replenishment (Kocagöz et al., 2022; Sireci et al., 2024; Calvo-Ochoa et al., 2025). Resolution of the proliferative response by 5 dph when the neuroepithelium is structurally and functionally restored, further indicates that this is a transient, injury-driven response.

Proliferating PCNA^+^ profiles in control fish were localized within the outer epithelial folds, where globose basal cells (GBCs) give rise to constitutive maintenance proliferation (Bayramli et al., 2017; Kocagöz et al., 2022). Following hypoxia, proliferating cells were found across the OE, including within the sensory region and the ILC, areas enriched with horizontal basal cells (HBCs), progenitors that contribute to damage-induced proliferation (Sireci et al., 2024). Concurrent with the peripheral proliferative response, we observed a robust increase in PCNA^+^ cells within the Vv region of the rostral telencephalon. Interestingly, proliferation in this region remained increased at 5 dph. The Vv is an active neurogenic niche densely packed with fast-cycling progenitors that constitutively give rise to proliferating neuroblasts that migrate toward the OB through a rostral migratory stream (RMS), similar to the one described in mammals (Adolf et al., 2006; Grandel et al., 2006; Kishimoto et al., 2011).

In contrast to OE proliferation, which subsides rapidly by 5 dph, the sustained activation of the Vv has important implications for supporting OB recovery. We posit that this prolonged response reflects the need for longer-term remodeling of OB neurons needed to replace olfactory glomerular connectivity disrupted by the transient loss of OSN afferents. While constitutive and postinjury neuronal replacement in the OE occurs quickly (Iqbal and Byrd-Jacobs, 2010; Kocagöz et al., 2022), newborn bulbar neurons generated in the Vv take several days to weeks to reach the OB (Kishimoto et al., 2011; Trimpe and Byrd-Jacobs, 2016; Godoy et al., 2020; Calvo-Ochoa et al., 2025). Furthermore, there were no changes in cell proliferation within the Vd region. This differential proliferation profile is consistent with the distinct progenitor identities of both proliferative niches: while the Vv is populated primarily by fast-cycling neuroblast progenitors, the Vd is composed mostly of slow-cycling progenitors whose output is directed toward telencephalic regions (Grandel et al., 2006; Kroehne et al., 2011; Rothenaigner et al., 2011; Kizil et al., 2012). The selective activation of Vv progenitors suggests that our sublethal hypoxic insult model may be insufficient to recruit slow-cycling populations that might respond to more severe injury signals to undergo proliferation. A study that used a comparable hypoxic exposure reported cell proliferation in the Vd domain. This discrepancy is likely explained by methodological differences: their hypoxic paradigm was considerably more severe, resulting in increased mortality; and proliferation was assessed by BrdU labeling and not PCNA (Lee et al., 2018). These differences suggest that the extent of brain damage associated with varying degrees of hypoxia severity is a determinant in the selective engagement of fast- versus slow-cycling progenitors in the telencephalon. Hypoxia regulates proliferation in the zebrafish nervous system via Wnt/β-catenin signaling (Meyers et al., 2012; Shitasako et al., 2017; Shimizu et al., 2018; Demirci et al., 2020) and, most notably, in the damaged OE (Kocagöz et al., 2022). Collectively, these findings indicate that the rapid recovery of olfactory function following hypoxic insult arises from a coordinated repair response that engages the proliferation of progenitor populations and the timely resolution of inflammatory signaling across the olfactory system.

An important consideration is that the mammalian olfactory system exhibits some repair and regenerative capabilities, although still limited when compared with those of zebrafish. Similar to zebrafish, the mammalian OE displays constitutive and postdamage OSN turnover through the activation of GBCs and HBCs, respectively (Schwob, 2002; Schwob et al., 2017). Another similarity lies in the constitutive replacement of OB interneurons though migration of neuroblasts from the hippocampal subventricular zone (SVZ; analogous to the zebrafish telencephalic VZ) though a RMS (Lois and Alvarez-Buylla, 1994). One of the major differences across species is the timescale at which this neuronal turnover occurs. In the mammalian OE, newborn OSNs innervate the OB in ∼4–6 weeks, while in zebrafish this occurs within 10–21 d (White et al., 2015; Calvo-Ochoa, 2025). In the rat, the timeline from constitutively neuroblast generation to OB integration is ∼3–6 weeks (Petreanu and Alvarez-Buylla, 2002; Carleton et al., 2003), while in zebrafish this process takes ∼2–3 weeks (Zupanc et al., 2005; Kishimoto et al., 2011). After OB dopaminergic interneuron loss, neuronal replacement can be observed by 1 month, while functional recovery is observed by 2 months postlesion (Lazarini et al., 2014), while in zebrafish structural and functional recovery is observed by 45 d in a similar lesion model. Importantly, there are differences in the mammalian OB repair response across lesion paradigms. Glutamatergic neuron loss in the mice OB does not stimulate compensatory neurogenesis in the SVZ, suggesting that the neurogenic response is tuned to dopaminergic loss (Liu and Guthrie, 2011). On the other hand, a similar experimental paradigm in zebrafish enhances the neurogenic response and leads to morphological recovery of the OB by 21 d (Calvo-Ochoa et al., 2025).

A feature that likely contributes to differences in the neuroregenerative abilities of zebrafish is inflammatory responses. While acute inflammation is the OE regulates proregenerative mechanisms in both models (Chen et al., 2017; Palominos et al., 2022), chronic inflammation prevents further OSN regeneration (Chen et al., 2019). In the mammalian OB, microglial activation impairs neurogenesis and the survival of newborn OB neurons (Lazarini et al., 2012), contrary to zebrafish, where microglial activation is necessary for neurogenesis and neuronal integration (Kyritsis et al., 2012; Caldwell et al., 2019). Additionally, the role of astroglia in regeneration presents divergent features. While mammalian astrogliosis that progresses to the formation of a glial scar hinders axonal regeneration (Cregg et al., 2014), zebrafish astroglia do not form glial scars (Scheib and Byrd-Jacobs, 2020). Furthermore, GFAP+ astroglial cells in the VZ are bona fide neural progenitors required for the neurogenic response (Kroehne et al., 2011; März et al., 2011; Baumgart et al., 2012). These fundamental differences in the role of inflammation in supporting neurogenesis and repair could lead to potential strategies to enhance postdamage olfactory recovery processes in mammalian models.

## Conclusions

This study provides the first comprehensive characterization of the cellular and morphological mechanisms underlying hypoxia-induced olfactory dysfunction. We extend prior work by revealing the structural basis for posthypoxic injury and repair across the olfactory axis in a model organism known for its remarkable regenerative capacity, the adult zebrafish. Further investigation of repair and regeneration processes described here may ultimately inform therapeutic strategies for enhancing neural repair following hypoxic–ischemic brain injury.

## Synthesis

Reviewing Editor: Arianna Maffei, Stony Brook University Decisions are customarily a result of the Reviewing Editor and the peer reviewers coming together and discussing their recommendations until a consensus is reached. When revisions are invited, a fact-based synthesis statement explaining their decision and outlining what is needed to prepare a revision will be listed below. The following reviewer(s) agreed to reveal their identity: Arianna Maffei.

The revised manuscript addressed most of the reviewers' concerns.

There are a few issues remaining:

1- Figure 1. The tank diagram in panel B covers part of the letter B and part of panel C.

2- Figure 1 panel G, the plot overlaps with the letter G making it unreadable

3-Similar issues apply to almost every panel in Figure 2

4- Figure 6, right/bottom panel, the first 2 images include a diagram of the location of the section which occupies too much on the image and include text that is unreadable.

5- Saying that fish are 'at least 6 months of age' is inadequate. If it is a large variance, then it would be important to ensure that there aren't large differences in ages of animals given different treatments. A bit more detail is still needed here.

## References

[B1] Adolf B, Chapouton P, Lam CS, Topp S, Tannhäuser B, Strähle U, Götz M, Bally-Cuif L (2006) Conserved and acquired features of adult neurogenesis in the zebrafish telencephalon. Dev Biol 295:278–293. 10.1016/j.ydbio.2006.03.023 16828638

[B2] Allen SP, Seehra RS, Heath PR, Hall BPC, Bates J, Garwood CJ, Matuszyk MM, Wharton SB, Simpson JE (2020) Transcriptomic analysis of human astrocytes in vitro reveals hypoxia-induced mitochondrial dysfunction, modulation of metabolism, and dysregulation of the immune response. Int J Mol Sci 21, 8028. 10.3390/ijms21218028 33126586 PMC7672558

[B3] Baumgart EV, Barbosa JS, Bally-Cuif L, Gotz M, Ninkovic J (2012) Stab wound injury of the zebrafish telencephalon: a model for comparative analysis of reactive gliosis. Glia 60:343–357. 10.1002/glia.22269 22105794

[B4] Bayramli X, Kocagöz Y, Sakizli U, Fuss SH (2017) Patterned arrangements of olfactory receptor gene expression in zebrafish are established by radial movement of specified olfactory sensory neurons. Sci Rep 7:5572. 10.1038/s41598-017-06041-1 28717156 PMC5514040

[B5] Bigdai E, Bezgacheva E, Samojlov V, Korolyev Y (2018) The effects of hypoxic hypoxia on olfactory sensitivity in humans. Biophysics 63:463–468. 10.1134/S0006350918030041

[B6] Bigdai EV, Bezgacheva EA, Vovenko EP, Samoilov VO (2019) Changes in the oxygen uptake rate in the rat olfactory epithelium under the influence of odorants. Biophysics 64:614–618. 10.1134/S0006350919040031

[B7] Binar M, Gokgoz MC (2021) Olfactory function in patients with obstructive sleep apnea and the effect of positive airway pressure treatment: a systematic review and meta-analysis. Sleep Breath 25:1791–1802. 10.1007/s11325-021-02349-5 33738753 PMC7972818

[B8] Braga MM, Rico EP, Córdova SD, Pinto CB, Blaser RE, Dias RD, Rosemberg DB, Oliveira DL, Souza DO (2013) Evaluation of spontaneous recovery of behavioral and brain injury profiles in zebrafish after hypoxia. Behav Brain Res 253:145–151. 10.1016/j.bbr.2013.07.008 23867150

[B9] Braga MM, et al. (2016) Brain zinc chelation by diethyldithiocarbamate increased the behavioral and mitochondrial damages in zebrafish subjected to hypoxia. Sci Rep 6:20279. 10.1038/srep20279 26854133 PMC4745017

[B10] Busl KM, Greer DM (2010) Hypoxic-ischemic brain injury: pathophysiology, neuropathology and mechanisms. NeuroRehabilitation 26:5–13. 10.3233/NRE-2010-0526 20130351

[B11] Byrd CA, Brunjes PC (1995) Organization of the olfactory system in the adult zebrafish: histological, immunohistochemical, and quantitative analysis. J Comp Neurol 358:247–259. 10.1002/cne.903580207 7560285

[B12] Cadiz L, Bundgaard A, Malte H, Fago A (2019) Hypoxia enhances blood O2 affinity and depresses skeletal muscle O2 consumption in zebrafish (*Danio rerio*). Comp Biochem Physiol Biochem Mol Biol 234:18–25. 10.1016/j.cbpb.2019.01.014 31075501

[B13] Caldwell LJ, Davies NO, Cavone L, Mysiak KS, Semenova SA, Panula P, Armstrong JD, Becker CG, Becker T (2019) Regeneration of dopaminergic neurons in adult zebrafish depends on immune system activation and differs for distinct populations. J Neurosci 39:4694–4713. 10.1523/JNEUROSCI.2706-18.2019 30948475 PMC6561686

[B14] Calvo-Ochoa E, Byrd-Jacobs CA, Fuss SH (2021) Diving into the streams and waves of constitutive and regenerative olfactory neurogenesis: insights from zebrafish. Cell Tissue Res 383:227–253. 10.1007/s00441-020-03334-2 33245413

[B15] Calvo-Ochoa E, Vorhees NW, Lockett TP, DeWitt-Batt SL, Thomas EA, Gray AB, Miyasaka N, Yoshihara Y, Byrd-Jacobs CA (2025) Structural regeneration and functional recovery of the olfactory system of adult zebrafish following brain injury. J Neurosci 45:e2456242025. 10.1523/JNEUROSCI.2456-24.2025 40789654 PMC12444855

[B16] Calvo-Ochoa E, Byrd-Jacobs CA (2019) The olfactory system of zebrafish as a model for the study of neurotoxicity and injury: implications for neuroplasticity and disease. Int J Mol Sci 20:1639. 10.3390/ijms20071639 30986990 PMC6480214

[B17] Carleton A, Petreanu LT, Lansford R, Alvarez-Buylla A, Lledo PM (2003) Becoming a new neuron in the adult olfactory bulb. Nat Neurosci 6:507–518. 10.1038/nn1048 12704391

[B18] Carrillo SA, Anguita-Salinas C, Pena OA, Morales RA, Munoz-Sanchez S, Munoz-Montecinos C, Paredes-Zúñiga S, Tapia K, Allende ML (2016) Macrophage recruitment contributes to regeneration of mechanosensory hair cells in the zebrafish lateral line. J Cell Biochem 117:1880–1889. 10.1002/jcb.25487 26755079

[B19] Chen M, Reed RR, Lane AP (2017) Acute inflammation regulates neuroregeneration through the NF-κB pathway in olfactory epithelium. Proc Natl Acad Sci U S A 114:8089–8094. 10.1073/pnas.1620664114 28696292 PMC5544262

[B20] Chen M, Reed RR, Lane AP (2019) Chronic inflammation directs an olfactory stem cell functional switch from neuroregeneration to immune defense. Cell Stem Cell 25:501–513.e5. 10.1016/j.stem.2019.08.011 31523027 PMC6778045

[B21] Chuah MI, West AK (2002) Cellular and molecular biology of ensheathing cells. Microsc Res Tech 58:216–227. 10.1002/jemt.10151 12203700

[B22] Cox JP (2008) Hydrodynamic aspects of fish olfaction. J R Soc Interface 5:575–593. 10.1098/rsif.2007.1281 18184629 PMC2408354

[B23] Cregg JM, DePaul MA, Filous AR, Lang BT, Tran A, Silver J (2014) Functional regeneration beyond the glial scar. Exp Neurol 253:197–207. 10.1016/j.expneurol.2013.12.024 24424280 PMC3951813

[B24] Das T, Soren K, Yerasi M, Kumar A, Chakravarty S (2019) Revealing sex-specific molecular changes in hypoxia-ischemia induced neural damage and subsequent recovery using zebrafish model. Neurosci Lett 712:134492. 10.1016/j.neulet.2019.134492 31518677

[B25] Demirci Y, Cucun G, Poyraz YK, Mohammed S, Heger G, Papatheodorou I, Ozhan G (2020) Comparative transcriptome analysis of the regenerating zebrafish telencephalon unravels a resource with key pathways during two early stages and activation of Wnt/β-catenin signaling at the early wound healing stage. Front Cell Dev Biol 8:584604. 10.3389/fcell.2020.584604 33163496 PMC7581945

[B26] Dhillon RS, Richards JG (2018) Hypoxia induces selective modifications to the acetylome in the brain of zebrafish (*Danio rerio*). Comp Biochem Physiol Biochem Mol Biol 224:79–87. 10.1016/j.cbpb.2017.12.018 29309913

[B27] Douglas RM, Ryu J, Kanaan A, Del Carmen Rivero M, Dugan LL, Haddad GG, Ali SS (2010) Neuronal death during combined intermittent hypoxia/hypercapnia is due to mitochondrial dysfunction. Am J Physiol Cell Physiol 298:C1594–C1602. 10.1152/ajpcell.00298.2009 20357179 PMC2889641

[B28] Esbaugh AJ, Perry SF, Gilmour KM (2009) Hypoxia-inducible carbonic anhydrase IX expression is insufficient to alleviate intracellular metabolic acidosis in the muscle of zebrafish, danio rerio. Am J Physiol Regul Integr Comp Physiol 296:R150–R160. 10.1152/ajpregu.90685.2008 18945954

[B29] Feng L, Han CX, Cao SY, Zhang HM, Wu GY (2020) Deficits in motor and cognitive functions in an adult mouse model of hypoxia-ischemia induced stroke. Sci Rep 10:20646. 10.1038/s41598-020-77678-8 33244072 PMC7692481

[B30] Franklin RJ, Barnett SC (1997) Do olfactory glia have advantages over Schwann cells for CNS repair? J Neurosci Res 50:665–672. 10.1002/(SICI)1097-4547(19971201)50:5<665::AID-JNR2>3.0.CO;2-P 9418955

[B31] Fuhrmann DC, Brüne B (2017) Mitochondrial composition and function under the control of hypoxia. Redox Biol 12:208–215. 10.1016/j.redox.2017.02.012 28259101 PMC5333533

[B32] Godoy R, Hua K, Kalyn M, Cusson VM, Anisman H, Ekker M (2020) Dopaminergic neurons regenerate following chemogenetic ablation in the olfactory bulb of adult zebrafish (*Danio rerio*). Sci Rep 10:12825. 10.1038/s41598-020-69734-0 32733000 PMC7393114

[B33] Grandel H, Kaslin J, Ganz J, Wenzel I, Brand M (2006) Neural stem cells and neurogenesis in the adult zebrafish brain: origin, proliferation dynamics, migration and cell fate. Dev Biol 295:263–277. 10.1016/j.ydbio.2006.03.040 16682018

[B34] Hansen A, Zielinski BS (2005) Diversity in the olfactory epithelium of bony fishes: development, lamellar arrangement, sensory neuron cell types and transduction components. J Neurocytol 34:183–208. 10.1007/s11068-005-8353-1 16841163

[B35] Harvie EA, Huttenlocher A (2015) Neutrophils in host defense: new insights from zebrafish. J Leukoc Biol 98:523–537. 10.1189/jlb.4RI0315-098R 25717145 PMC4569048

[B36] He S-M, Sun S, Chen A-Q, Lv S-J, Qiu C-Z, Wei M-L, Liu W, Liu HR, Zhang L, Ren D-L (2022) Hypoxia regulates cytokines expression and neutrophils migration by ERK signaling in zebrafish. Fish Shellfish Immunol 125:212–219. 10.1016/j.fsi.2022.04.043 35569778

[B37] Hentig JT, Byrd-Jacobs CA (2016) Exposure to zinc sulfate results in differential effects on olfactory sensory neuron subtypes in adult zebrafish. Int J Mol Sci 17:1445. 10.3390/ijms17091445 27589738 PMC5037724

[B38] Hernández-Soto R, Villasana-Salazar B, Pinedo-Vargas L, Peña-Ortega F (2021) Chronic intermittent hypoxia alters main olfactory bulb activity and olfaction. Exp Neurol 340:113653. 10.1016/j.expneurol.2021.113653 33607078

[B39] Huppertz T, Freiherr J, Olzowy B, Kisser U, Stephan J, Fesl G, Haegler K, Feddersen B, Fischer R, Mees K, Mees K (2018) Reduction of olfactory sensitivity during normobaric hypoxia. Auris Nasus Larynx 45:747–752. 10.1016/j.anl.2017.09.004 29153259

[B40] Hussain A, Saraiva LR, Ferrero DM, Ahuja G, Krishna VS, Liberles SD, Korsching SI (2013) High-affinity olfactory receptor for the death-associated odor cadaverine. Proc Natl Acad Sci U S A 110:19579–19584. 10.1073/pnas.1318596110 24218586 PMC3845148

[B41] Iqbal T, Byrd-Jacobs C (2010) Rapid degeneration and regeneration of the zebrafish olfactory epithelium after triton X-100 application. Chem Senses 35:351–361. 10.1093/chemse/bjq019 20228140 PMC2871777

[B42] Kaslin J, Ganz J, Brand M (2008) Proliferation, neurogenesis and regeneration in the non-mammalian vertebrate brain. Philos Trans R Soc Lond B Biol Sci 363:101–122. 10.1098/rstb.2006.2015 17282988 PMC2605489

[B43] Kermen F, Darnet L, Wiest C, Palumbo F, Bechert J, Uslu O, Yaksi E (2020) Stimulus-specific behavioral responses of zebrafish to a large range of odors exhibit individual variability. BMC Biol 18:1–16. 10.1186/s12915-020-00873-0 32539727 PMC7296676

[B44] Kim B-Y, Park JY, Bae JH (2023) The effects of the levels of hypoxia in the olfactory nervous system in mouse model. Am J Rhinol Allergy 37:575–585. 10.1177/19458924231176440 37350017

[B45] Kishimoto N, Alfaro-Cervello C, Shimizu K, Asakawa K, Urasaki A, Nonaka S, Kawakami K, Garcia-Verdugo JM, Sawamoto K (2011) Migration of neuronal precursors from the telencephalic ventricular zone into the olfactory bulb in adult zebrafish. J Comp Neurol 519:3549–3565. 10.1002/cne.22722 21800305

[B46] Kizil C, Kaslin J, Kroehne V, Brand M (2012) Adult neurogenesis and brain regeneration in zebrafish. Dev Neurobiol 72:429–461. 10.1002/dneu.20918 21595047

[B47] Kocagöz Y, Demirler MC, Eski SE, Güler K, Dokuzluoglu Z, Fuss SH (2022) Disparate progenitor cell populations contribute to maintenance and repair neurogenesis in the zebrafish olfactory epithelium. Cell Tissue Res 388:331–358. 10.1007/s00441-022-03598-4 35266039

[B48] Kroehne V, Freudenreich D, Hans S, Kaslin J, Brand M (2011) Regeneration of the adult zebrafish brain from neurogenic radial glia-type progenitors. Development 138:4831–4841. 10.1242/dev.072587 22007133

[B49] Kyritsis N, Kizil C, Zocher S, Kroehne V, Kaslin J, Freudenreich D, Freudenreich D, Iltzsche A, Brand M (2012) Acute inflammation initiates the regenerative response in the adult zebrafish brain. Science 338:1353–1356. 10.1126/science.1228773 23138980

[B50] Lazarini F, Gabellec MM, Torquet N, Lledo PM (2012) Early activation of microglia triggers long-lasting impairment of adult neurogenesis in the olfactory bulb. J Neurosci 32:3652–3664. 10.1523/JNEUROSCI.4394-11.2012 22423088 PMC6703455

[B51] Lazarini F, Gabellec MM, Moigneu C, de Chaumont F, Olivo-Marin JC, Lledo PM (2014) Adult neurogenesis restores dopaminergic neuronal loss in the olfactory bulb. J Neurosci 34:14430–14442. 10.1523/JNEUROSCI.5366-13.2014 25339754 PMC6608394

[B52] Lazzari M, Bettini S, Franceschini V (2014) Immunocytochemical characterisation of olfactory ensheathing cells of zebrafish. J Anat 224:192–206. 10.1111/joa.12129 24164558 PMC3969062

[B53] Lecoq J, Tiret P, Najac M, Shepherd GM, Greer CA, Charpak S (2009) Odor-evoked oxygen consumption by action potential and synaptic transmission in the olfactory bulb. J Neurosci 29:1424–1433. 10.1523/JNEUROSCI.4817-08.2009 19193889 PMC2662132

[B54] Lee Y, Lee S, Park J-W, Hwang J-S, Kim S-M, Lyoo IK, Lee C-J, Han I-O (2018) Hypoxia-induced neuroinflammation and learning–memory impairments in adult zebrafish are suppressed by glucosamine. Mol Neurobiol 55:8738–8753. 10.1007/s12035-018-1017-9 29589284

[B55] Leu T, Schutzhold V, Fandrey J, Ferenz KB (2019) When the brain yearns for oxygen. Neurosignals 27:50–61. 10.33594/000000199 31860206

[B56] Liu H, Guthrie KM (2011) Neuronal replacement in the injured olfactory bulb. Exp Neurol 228:270–282. 10.1016/j.expneurol.2011.01.021 21310147 PMC3063445

[B57] Lois C, Alvarez-Buylla A (1994) Long-distance neuronal migration in the adult mammalian brain. Science 264:1145–1148. 10.1126/science.8178174 8178174

[B58] Marina N, Kasymov V, Ackland GL, Kasparov S, Gourine AV (2016) Astrocytes and brain hypoxia. Adv Exp Med Biol 903:201–207. 10.1007/978-1-4899-7678-9_14 27343098

[B59] Marino KM, Silva ER, Windelborn JA (2020) A comparison between chemical and gas hypoxia as models of global ischemia in zebrafish (*Danio rerio*). Anim Models Exp Med 3:256–263. 10.1002/ame2.12133 PMC7529334 33024947

[B60] März M, Chapouton P, Diotel N, Vaillant C, Hesl B, Takamiya M, Lam CS, Kah O, Bally-Cuif L, Strähle U (2010) Heterogeneity in progenitor cell subtypes in the ventricular zone of the zebrafish adult telencephalon. Glia 58:870–888. 10.1002/glia.20971 20155821

[B61] März M, Schmidt R, Rastegar S, Strähle U (2011) Regenerative response following stab injury in the adult zebrafish telencephalon. Dev Dyn 240:2221–2231. 10.1002/dvdy.22710 22016188

[B62] Masuda M, Ihara S, Mori N, Koide T, Miyasaka N, Wakisaka N, Yoshikawa K, Watanabe H, Touhara K, Yoshihara Y (2024) Identification of olfactory alarm substances in zebrafish. Curr Biol 34:1377–1389.e1377. 10.1016/j.cub.2024.02.003 38423017

[B63] Merelli A, Repetto M, Lazarowski A, Auzmendi J (2021) Hypoxia, oxidative stress, and inflammation: three faces of neurodegenerative diseases. J Alzheimers Dis 82:S109–S126. 10.3233/jad-201074 33325385

[B64] Meyers JR, Hu L, Moses A, Kaboli K, Papandrea A, Raymond PA (2012) β-catenin/Wnt signaling controls progenitor fate in the developing and regenerating zebrafish retina. Neural Dev 7:30. 10.1186/1749-8104-7-30 22920725 PMC3549768

[B65] Miyasaka N, Morimoto K, Tsubokawa T, Higashijima S, Okamoto H, Yoshihara Y (2009) From the olfactory bulb to higher brain centers: genetic visualization of secondary olfactory pathways in zebrafish. J Neurosci 29:4756–4767. 10.1523/JNEUROSCI.0118-09.2009 19369545 PMC6665349

[B66] Mokhtar DM, Zaccone G, Alesci A, Kuciel M, Hussein MT, Sayed RK (2023) Main components of fish immunity: an overview of the fish immune system. Fishes 8:93. 10.3390/fishes8020093

[B67] Mori K, Nagao H, Yoshihara Y (1999) The olfactory bulb: coding and processing of odor molecule information. Science 286:711–715. 10.1126/science.286.5440.711 10531048

[B68] Nawroth JC, Greer CA, Chen WR, Laughlin SB, Shepherd GM (2007) An energy budget for the olfactory glomerulus. J Neurosci 27:9790–9800. 10.1523/JNEUROSCI.1415-07.2007 17804639 PMC6672954

[B69] Nunez-Parra A, Cortes-Campos C, Bacigalupo J, Garcia MeL, Nualart F, Reyes JG (2011) Expression and distribution of facilitative glucose (GLUTs) and monocarboxylate/H+ (MCTs) transporters in rat olfactory epithelia. Chem Senses 36:771–780. 10.1093/chemse/bjr052 21677031

[B70] Ogawa K, et al. (2021) Frontline science: conversion of neutrophils into atypical Ly6G+ SiglecF+ immune cells with neurosupportive potential in olfactory neuroepithelium. J Leukoc Biol 109:481–496. 10.1002/JLB.3HI0620-410R 32725843

[B71] Palominos MF, Calfún C, Nardocci G, Candia D, Torres-Paz J, Whitlock KE (2022) The olfactory organ is a unique site for neutrophils in the brain. Front Immunol 13:881702. 10.3389/fimmu.2022.881702 35693773 PMC9186071

[B72] Park J, Jung S, Kim SM, Park IY, Bui NA, Hwang GS, Han IO (2021) Repeated hypoxia exposure induces cognitive dysfunction, brain inflammation, and amyloid-β/p-Tau accumulation through reduced brain O-GlcNAcylation in zebrafish. J Cereb Blood Flow Metab 41:3111–3126. 10.1177/0271678x211027381 34176340 PMC8756468

[B73] Pellegrini E, Mouriec K, Anglade I, Menuet A, Le Page Y, Gueguen MM, Marmignon M-H, Brion F, Pakdel F, Kah O (2007) Identification of aromatase-positive radial glial cells as progenitor cells in the ventricular layer of the forebrain in zebrafish. J Comp Neurol 501:150–167. 10.1002/cne.21222 17206614

[B74] Petreanu L, Alvarez-Buylla A (2002) Maturation and death of adult-born olfactory bulb granule neurons: role of olfaction. J Neurosci 22:6106–6113. 10.1523/JNEUROSCI.22-14-06106.2002 12122071 PMC6757952

[B75] Polosukhin VV, Cates JM, Lawson WE, Milstone AP, Matafonov AG, Massion PP, Lee JW, Randell SH, Blackwell TS (2011) Hypoxia-inducible factor-1 signalling promotes goblet cell hyperplasia in airway epithelium. J Pathol 224:203–211. 10.1002/path.2863 21557221 PMC5901746

[B76] Raisman G, Li Y (2007) Repair of neural pathways by olfactory ensheathing cells. Nat Rev Neurosci 8:312–319. 10.1038/nrn2096 17342173

[B77] Rodriguez A, Zhang H, Klaminder J, Brodin T, Andersson PL, Andersson M (2018) Toxtrac: a fast and robust software for tracking organisms. Methods Ecol Evol 9:460–464. 10.1111/2041-210X.12874

[B78] Rothenaigner I, Krecsmarik M, Hayes JA, Bahn B, Lepier A, Fortin G, Fortin G, Götz M, Jagasia R, Bally-Cuif L (2011) Clonal analysis by distinct viral vectors identifies bona fide neural stem cells in the adult zebrafish telencephalon and characterizes their division properties and fate. Development 138:1459–1469. 10.1242/dev.058156 21367818

[B79] Rygg AD, van Duin AC, Craven BA (2013) Molecular dynamics simulations of water/mucus partition coefficients for feeding stimulants in fish and the implications for olfaction. PLoS One 8:e72271. 10.1371/journal.pone.0072271 24023732 PMC3759373

[B80] Saraiva LR, Ahuja G, Ivandic I, Syed AS, Marioni JC, Korsching SI, Logan DW (2015) Molecular and neuronal homology between the olfactory systems of zebrafish and mouse. Sci Rep 5:11487. 10.1038/srep11487 26108469 PMC4480006

[B81] Sato Y, Miyasaka N, Yoshihara Y (2005) Mutually exclusive glomerular innervation by two distinct types of olfactory sensory neurons revealed in transgenic zebrafish. J Neurosci 25:4889–4897. 10.1523/jneurosci.0679-05.2005 15901770 PMC6724860

[B82] Scheib J, Byrd-Jacobs C (2020) Zebrafish astroglial morphology in the olfactory bulb is altered with repetitive peripheral damage. Front Neuroanat 14:4. 10.3389/fnana.2020.00004 32116575 PMC7026507

[B83] Schwob JE (2002) Neural regeneration and the peripheral olfactory system. Anat Rec 269:33–49. 10.1002/ar.10047 11891623

[B84] Schwob JE, Jang W, Holbrook EH, Lin B, Herrick DB, Peterson JN, Coleman JH (2017) Stem and progenitor cells of the mammalian olfactory epithelium: taking poietic license. J Comp Neurol 525:1034–1054. 10.1002/cne.24105 27560601 PMC5805156

[B85] Scultetus AH, et al. (2016) Brain hypoxia is exacerbated in hypobaria during aeromedical evacuation in swine with traumatic brain injury. J Trauma Acute Care Surg 81:101–107. 10.1097/ta.0000000000001048 26998778

[B86] Shimizu Y, Ueda Y, Ohshima T (2018) Wnt signaling regulates proliferation and differentiation of radial glia in regenerative processes after stab injury in the optic tectum of adult zebrafish. Glia 66:1382–1394. 10.1002/glia.23311 29411422

[B87] Shitasako S, Ito Y, Ito R, Ueda Y, Shimizu Y, Ohshima T (2017) Wnt and Shh signals regulate neural stem cell proliferation and differentiation in the optic tectum of adult zebrafish. Dev Neurobiol 77:1206–1220. 10.1002/dneu.22509 28589698

[B88] Simonis C, Zink L, Johnston SE, Bogard M, Pyle GG (2024) Effects of water quality on palladium-induced olfactory toxicity and bioaccumulation in rainbow trout (oncorhynchus mykiss). Integr Environ Assess Manag 20:1407–1419. 10.1002/ieam.4900 38329152

[B89] Sireci S, Kocagöz Y, Alkiraz AS, Güler K, Dokuzluoglu Z, Balcioglu E, Meydanli S, Demirler MC, Erdogan NS, Fuss SH (2024) HB-EGF promotes progenitor cell proliferation and sensory neuron regeneration in the zebrafish olfactory epithelium. FEBS J 291:2098–2133. 10.1111/febs.17033 38088047

[B90] Skaggs K, Goldman D, Parent JM (2014) Excitotoxic brain injury in adult zebrafish stimulates neurogenesis and long-distance neuronal integration. Glia 62:2061–2079. 10.1002/glia.22726 25043622 PMC4205181

[B91] Tigert LR, Hubbard PC, Porteus CS (2025) Effects of hypoxia on the olfactory sensitivity of gilt-head seabream (*Sparus aurata*). J Exp Biol 228:jeb249771. 10.1242/jeb.249771 39555894 PMC11744318

[B92] Trimpe DM, Byrd-Jacobs CA (2016) Patterns of olfactory bulb neurogenesis in the adult zebrafish are altered following reversible deafferentation. Neuroscience 331:134–147. 10.1016/j.neuroscience.2016.06.026 27343831 PMC6496944

[B93] Tsarouchas TM, et al. (2018) Dynamic control of proinflammatory cytokines Il-1β and Tnf-α by macrophages in zebrafish spinal cord regeneration. Nat Commun 9:4670. 10.1038/s41467-018-07036-w 30405119 PMC6220182

[B94] Van Ginneken V, van den Thillart G, Addink A, Erkelens C (1995) Fish muscle energy metabolism measured during hypoxia and recovery: an in vivo 31P-NMR study. Am J Physiol Regul Integr Comp Physiol 268:R1178–R1187. 10.1152/ajpregu.1995.268.5.R1178 7771577

[B95] Var SR, Byrd-Jacobs CA (2020) Role of macrophages and microglia in zebrafish regeneration. Int J Mol Sci 21:24768. 10.3390/ijms21134768 PMC7369716 32635596

[B96] Villar PS, Delgado R, Vergara C, Reyes JG, Bacigalupo J (2017) Energy requirements of odor transduction in the chemosensory cilia of olfactory sensory neurons rely on oxidative phosphorylation and glycolytic processing of extracellular glucose. J Neurosci 37:5736–5743. 10.1523/JNEUROSCI.2572-16.2017 28500222 PMC6596473

[B97] Villegas R, Martin SM, O’Donnell KC, Carrillo SA, Sagasti A, Allende ML (2012) Dynamics of degeneration and regeneration in developing zebrafish peripheral axons reveals a requirement for extrinsic cell types. Neural Dev 7:19. 10.1186/1749-8104-7-19 22681863 PMC3780720

[B98] Vorhees NW, Groenwold SL, Williams MT, Putt LS, Sanchez-Gama N, Stalions GA, Taylor GM, Van Dort HE, Calvo-Ochoa E (2025) Olfactory dysfunction in a novel model of prodromal Parkinson’s disease in adult zebrafish. Int J Mol Sci 26, 4474. 10.3390/ijms26104474 40429620 PMC12111043

[B99] Walmsley SR, et al. (2005) Hypoxia-induced neutrophil survival is mediated by HIF-1alpha-dependent NF-kappaB activity. J Exp Med 201:105–115. 10.1084/jem.20040624 15630139 PMC2212759

[B100] Wang X, Cui L, Ji X (2022) Cognitive impairment caused by hypoxia: from clinical evidences to molecular mechanisms. Metab Brain Dis 37:51–66. 10.1007/s11011-021-00796-3 34618295

[B101] White EJ, Kounelis SK, Byrd-Jacobs CA (2015) Plasticity of glomeruli and olfactory-mediated behavior in zebrafish following detergent lesioning of the olfactory epithelium. Neuroscience 284:622–631. 10.1016/j.neuroscience.2014.10.024 25450960 PMC4267997

[B102] Williams CR, Dittman AH, McElhany P, Busch DS, Maher MT, Bammler TK, MacDonald JW, Gallagher EP (2019) Elevated CO2 impairs olfactory-mediated neural and behavioral responses and gene expression in ocean-phase coho salmon (*Oncorhynchus kisutch*). Glob Chang Biol 25:963–977. 10.1111/gcb.14532 30561876 PMC7065673

[B103] Yan EB, Satgunaseelan L, Paul E, Bye N, Nguyen P, Agyapomaa D, Kossmann T, Rosenfeld JV, Morganti-Kossmann MC (2014) Post-traumatic hypoxia is associated with prolonged cerebral cytokine production, higher serum biomarker levels, and poor outcome in patients with severe traumatic brain injury. J Neurotrauma 31:618–629. 10.1089/neu.2013.3087 24279428 PMC3961772

[B104] Yoshihara Y (2009) Molecular genetic dissection of the zebrafish olfactory system. Results Probl Cell Differ 47:97–120. 10.1007/400_2008_1 19083130

[B105] Yu X, Li YV (2011) Zebrafish as an alternative model for hypoxic-ischemic brain damage. Int J Physiol Pathophysiol Pharmacol 3:88–96. 21760967 PMC3134003

[B106] Zupanc GKH, Hinsch K, Gage FH (2005) Proliferation, migration, neuronal differentiation, and long-term survival of new cells in the adult zebrafish brain. J Comp Neurol 488:290–319. 10.1002/cne.20571 15952170

